# Molecular characteristics and clinical implications of serine/arginine-rich splicing factors in human cancer

**DOI:** 10.18632/aging.205241

**Published:** 2023-11-24

**Authors:** Jinjin Zhang, Zhicheng Fang, Congkuan Song

**Affiliations:** 1 Department of Emergency Medicine, Taihe Hospital, Hubei University of Medicine, Shiyan, China; 2 Department of Thoracic Surgery, Renmin Hospital of Wuhan University, Wuhan, China

**Keywords:** serine/arginine-rich splicing factors, pan-cancer, genomic alterations, cancer immunity, oncogenic pathways

## Abstract

As critical splicing regulators, serine/arginine-rich splicing factors (SRSFs) play pivotal roles in carcinogenesis. As dysregulation of SRSFs may confer potential cancer risks, targeting SRSFs could provide important insights into cancer therapy. However, a global and comprehensive pattern to elaborate the molecular characteristics, mechanisms, and clinical links of SRSFs in a wide variety of human cancer is still lacking. In this study, a systematic analysis was conducted to reveal the molecular characteristics and clinical implications of SRSFs covering more than 10000 tumour samples of 33 human cancer types. We found that SRSFs experienced prevalent genomic alterations and expression perturbations in multiple cancer types. The DNA methylation, m6A modification, and miRNA regulation of SRSFs were all cancer context-dependent. Importantly, we found that SRSFs were strongly associated with cancer immunity, and were capable of predicting response to immunotherapy. And SRSFs had colossal potential for predicting survival in multiple cancer types, including those that have received immunotherapy. Moreover, we also found that SRSFs could indicate the drug sensitivity of targeted therapy and chemotherapy. Our research highlights the significance of SRSFs in cancer occurrence and development, and provides sufficient resources for understanding the biological characteristics of SRSFs, offering a new and unique perspective for developing cancer therapeutic strategies.

## INTRODUCTION

It is estimated that about 90% of human pre-mRNA undergo alternative splicing events [1, 2]. However, the abnormal regulation of RNA alternative splicing is a crucial process in carcinogenesis [3]. RNA alternative splicing regulators play essential roles in this process and can act as oncogenes, leading to cancer progression and metastasis by producing some RNA subtypes in key oncogenic pathways [4–6].

**Figure 1:**
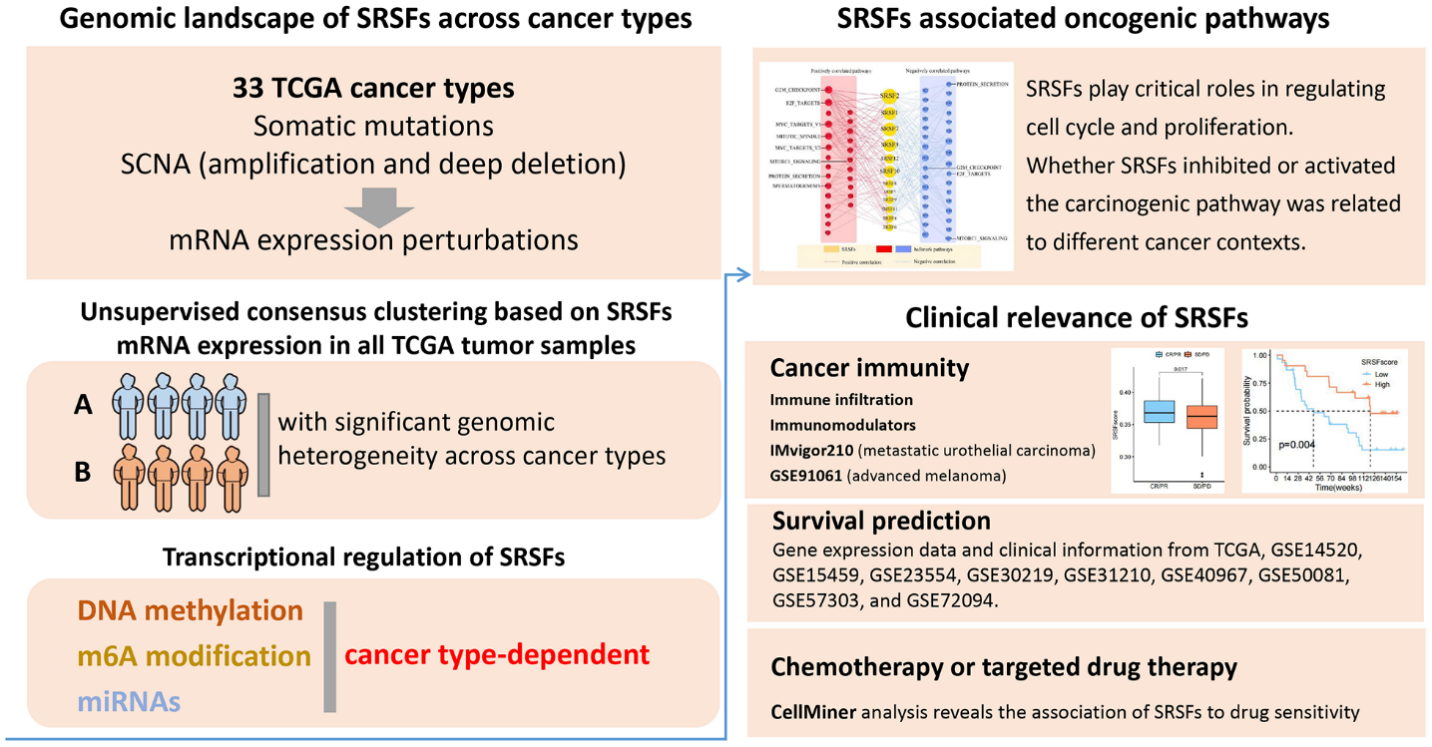
Workflow chart of this study.

Serine/arginine-rich splicing factors (SRSFs) are important splicing regulators [7], usually consisting of 12 members (SRSF1-12), which play pivotal roles in mRNA’s turnover, output, and various post-transcriptional regulation of other splices [8]. Dysregulation of SRSFs expression is closely related to carcinogenesis. It can lead to the change of alternative splicing patterns of essential genes [3, 9, 10]; On the other hand, it can also significantly disrupt genomic stability, resulting in abnormal biological function [11, 12]. However, a global and comprehensive pattern to elaborate the molecular characteristics, mechanisms, and clinical links of SRSFs in multiple human cancer is still lacking. Here, we attempted to systematically uncover the molecular features and clinical implications of SRSFs from various levels, including genome (somatic mutation, copy number alteration), mRNA expression, transcriptional regulation (DNA methylation, N6-methyladenosine (m6A) modification, miRNA), immune links, survival prediction and drug sensitivity, in more than 10000 patients across 33 cancer types. Our comprehensive analysis highlights the pivotal roles of SRSFs in cancer occurrence and development. This study provides rich resources for understanding the biological characteristics of SRSFs, offering a new and unique perspective for developing cancer therapeutic strategies based on RNA alternative splicing. The flowchart and important findings of this study were showed in Figure 1.

## MATERIALS AND METHODS

### Genomic, transcriptomic data acquisition and analysis

Somatic mutation data and TCGA threshold SCNA scores from 33 human cancer types were obtained from the Genomic Data Commons (https://gdc.cancer.gov/) and processed as previously described [13–16]. Our study defined copy number variation (CNV) amplification and deletion in the same way, as described by Knijnenburg et al. [17]. The frequency of genomic alterations (including mutation and SCNA) of specific genes in pan-cancer were shown as heatmaps. We also obtained the relevant data for genomic instability scores, including the mutation burden, the aneuploidy score, the SCNA burden, loss of heterozygosity (LOH) score and homologous recombination deficiency (HRD) score from the study of Knijnenburg et al. [17], and defined these scores as previously described [15, 17]. Additionally, we obtained the batch effects-corrected TCGA mRNA data (FPKM-based gene expression) and clinical information (including the survival status and survival time) for patients across 33 cancer types from TCGA Genomic Data Commons.

### Differential expression analysis and unsupervised consensus clustering

19 cancer types, with at least 3 matched tumours and normal samples, were selected out of 33 cancer types for differential expression analysis. Differentially expressed genes (DEGs) were identified by Wilcox’s rank sum test, and the p-values were adjusted by BH method. We defined the DEGs as those whose expression differences were associated with adjusted p-values < 0.05. Additionally, unsupervised consensus clustering was performed to identify distinct patient clusters based on SRSFs mRNA expression in all tumor samples across 33 cancer types. The “ConsensuClusterPlus” R package [18] was used to carry out the steps above. To guarantee classification stability, 1000-time repetitions were conducted.

### DNA methylation, m6A modification and miRNA analysis

DNA methylation data files across 33 cancer types were retrieved from the Genomic Data Commons. We processed this data as previously described [15]. We calculated median beta value of these 12 SRSFs in each sample for better assessment of their overall methylation levels. Subsequently, Pearson correlation analysis between DNA methylation beta values and mRNA expression values for each SRSF was conducted for deeper investigation of the regulation of SRSFs expression by DNA methylation (| Cor |> 0 and *P*-value < 0.05 was set as the threshold). Also, 23 m6A regulators (including 13 readers, 8 writers and 2 erasers) were collected from previously published papers [19–21]. As the most common type of RNA modification, m6A plays an essential role in the occurrence and development of tumors [22]. We investigated the Pearson correlation coefficients between m6A regulators and SRSFs in 33 cancer types to further investigate the regulation of SRSFs expression by m6A methylation. Additionally, normalized miRNA expression data were downloaded from the Genomic Data Commons. We calculated the Pearson correlation coefficients between miRNAs and SRSFs in 33 cancer types to further investigate the regulation of SRSFs expression by miRNA. We also screened the preliminary results, as in the method of Luo et al. [15]. By setting the more stringent criteria, we further filtered the miRNAs acting with these SRSFs. The specific criteria were as follows: 1) |Pearson correlation coefficient| ≥ 0.25 and p < 0.05; 2) In at least one-third of all cancer types (at least ten cancer types), this miRNA acts on the same SRSF. This miRNA-mRNA interaction network was displayed using Cytoscape v3.9.0. The interrelationship between miRNAs and SRSFs in specific cancer types was also shown in a heatmap.

### Biological pathway activity across cancer types

FPKM-based gene expression was converted into Z-score using the “zFPKM” R package [23] for assessing the activity of 50 hallmark pathways. Gene Set Variation Analysis (GSVA) was performed [24]. In addition, Pearson correlation coefficients between mRNA expression of SRSFs and pathway activity were calculated to identify the SRSFs related to pathway activation or inhibition. And SRSF-pathway pairs were identified by the criterion we set (|Pearson correlation coefficient| ≥ 0.20 and p < 0.05). Genes do not function independently. To infer the overall SRSFs activity, “SRSFscore” was calculated based on 12 SRSFs mRNA expression within each cancer type as previously described [15, 25, 26]. Identification of biological pathways associated with the SRSFscore was also performed in the same way as in Luo et al. [15]. Here, we reported only pathways that showed consistent significant correlations (q-value < 0.05) in at least 10 cancer types.

### Immune correlation analysis

The Xcell algorithm [27] was used to quantify the infiltrating abundance of immune cell and tumor microenvironment scores for all tumor samples. We obtained a list of immunomodulators that are crucial in immunotherapy from previous publications [14]. To investigate the interconnection between cancer immunity and SRSFs, we examined the Pearson correlation coefficients between mRNA expression of immunomodulators or abundance of immune cells and the SRSFscore across 33 cancer types. Additionally, in order to explore the association between SRSFscore and the effect of immunotherapy as well as prognosis in patients receiving anti-PD-1 or anti-CTLA4 treatment, we further obtained the transcriptome data before immunotherapy and clinical information from two cancer immunotherapy cohorts [28, 29]. In our study, responders to immunotherapy included the patients with complete and partial remission (CR/PR), while non-responders included the patients with stable disease (SD) and progressive disease (PD). Wilcox’s rank sum test was applied to compare the SRSFscore between the two groups (responders and non-responders).

### Survival analysis

TCGA pan-cancer clinical data were downloaded from Genomic Data Commons. We used the “maxstat” R package to determine the best cut point for the SRSFscore, and divided the patients into high and low SRSFscore groups. Probability of overall survival was estimated by the Kaplan-Meier method, with differences between two groups tested using the log-rank test. Using the “survival” R package, we also performed Cox regression analysis to test the association between SRSFs expression and survival. Moreover, to validate the potential of SRSFscore in pan-cancer survival prediction, from the Gene Expression Omnibus (https://www.ncbi.nlm.nih.gov/geo/), we also downloaded gene expression data and corresponding clinical information for 9 gene chips (GSE14520 [30, 31], GSE15459 [32–34], GSE23554 [35], GSE30219 [36], GSE31210 [37, 38], GSE40967 [39], GSE50081 [40], GSE57303 [41], GSE72094 [42]) including 5 cancer types (OV, LUAD, LIHC, STAD and COAD) (Table 1). According to the corresponding annotation files, we converted the probes to gene symbols. For genes with multiple probe set signals, their values were averaged to generate a single expression value. In each independent dataset, we calculated the SRSFscore for each sample in the same way and investigated its relationship with the prognosis.

**Table 1:** Information on the nine GEO datasets included in this study.

**Datasets**	**Cancer types**	**No. of patients**	**Platforms**
GSE14520	LIHC	N = 21	GPL571
GSE15459	STAD	N = 192	GPL570
GSE23554	OV	N = 28	GPL96
GSE30219	LUAD	N = 85	GPL570
GSE31210	LUAD	N = 226	GPL570
GSE40967	COAD	N = 556	GPL570
GSE50081	LUAD	N = 127	GPL570
GSE57303	STAD	N = 70	GPL570
GSE72094	LUAD	N = 398	GPL15048

### CellMiner analysis

We downloaded the RNA data (RNA: RNA-seq) of 22379 genes identified in NCI-60 cell lines and the related data of 20503 compounds analyzed (compound activity: DTP NCI-60) from CellMiner (https://discover.nci.nih.gov/cellminer/home.do) [43]. We investigated the Pearson correlation coefficients between Z scores of clinical trials and FDA-approved drugs and mRNA expression values for each SRSF. We reported drug-SRSF pairs showing significant correlation (|Pearson correlation coefficient| ≥ 0.3 and *P*-value < 0.01).

### Statistical analysis

All of the analyses were performed in the R 3.6.3 software. A *P*-value < 0.05 was considered statistically different.

### Data availability

The datasets generated and analysed during the current study are available in the TCGA GDC repository, (https://portal.gdc.cancer.gov), GEO repository, (https://www.ncbi.nlm.nih.gov/geo/), and CellMiner repository, (https://discover.nci.nih.gov/cellminer/home.do).

## RESULTS

### SRSFs experienced prevalent genomic alterations and expression perturbations in multiple cancer types

**Figure 2:**
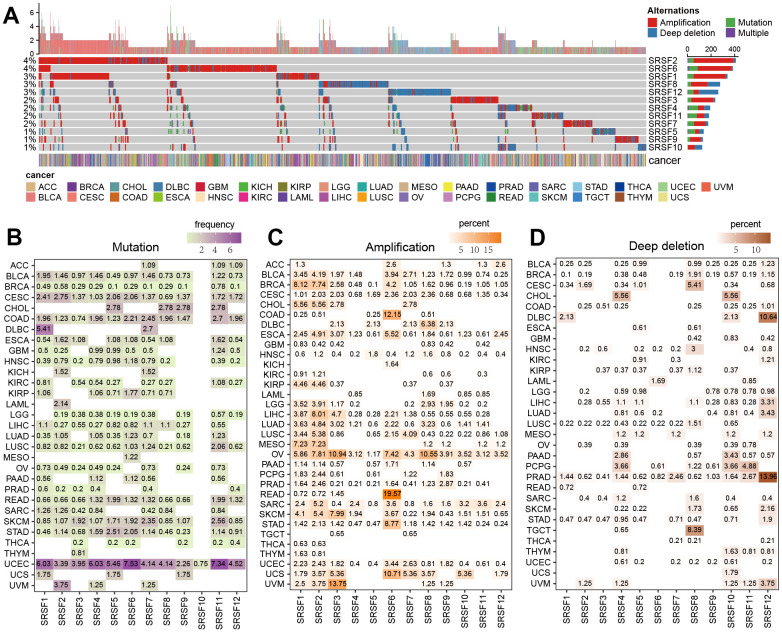
**The genomic alterations of SRSFs in human cancer.** (**A**) Genomic alterations [non-silent mutation and SCNA] landscape in the SRSFs in 33 cancer types. (**B**) Distribution of mutation frequencies across cancer types. (**C**, **D**) Distribution of SCNA (**C**: CNV amplification; **D**: CNV deep deletion) frequencies across cancer types. The darkness of color is proportional to the frequency.

As previously described [44], genomic alteration was defined as somatic mutations and SCNA (amplification and deep deletion). We first determined the overall genomic alteration level of SRSFs in human cancer. The frequencies were very low, only between 1% and 4% (Figure 2A). Among the 12 SRSFs, SRSF2 and SRSF6 displayed the highest alterations (4%), mostly from CNV amplification. While SRSF5, SRSF9 and SRSF10 were in the opposite situations (Figure 2A). SCNA counted for the majority of genomic alterations. Thus, we speculated that at the genomic level, it was SCNA, especially CNV amplification, but not mutation, which was the major cause of SRSFs dysregulation in cancer.

From an in-depth exploration of the genomic alteration pattern across 33 cancer types, we observed a low overall average mutation frequency of SRSFs from 0 - 7.53%. Uterine Corpus Endometrial Carcinoma (UCEC), with a higher global mutation burden [45], presented higher mutation frequencies in all 12 SRSFs, while Testicular Germ Cell Tumors (TGCT) and Pheochromocytoma and Paraganglioma (PCPG) lacked any mutation (Figure 2B). SRSF10 was slightly mutated (0.75%) only in UCEC, but not in other cancer types. Analogously, in LAMA, only SRSF2 experienced somatic mutation (2.14%). We noted that individual SRSF exhibited a cancer-type-dependent CNV amplification or deep deletion pattern (Figure 2C, 2D). For example, SRSF1 and SRSF2 presented relatively higher CNV amplification in the vast majority of cancer types, with the lack of deep deletion. SRSF12 showed higher deep deletion in DLBC (10.64%) and PRAD (13.96%) (Figure 2D). Interestingly, among these SRSFs, SRSF6 displayed higher amplification in digestive tract tumors, such as ESCA (5.52%), STAD (8.77%), COAD (12.15%) and READ (19.57%) (Figure 2C).

**Figure 3:**
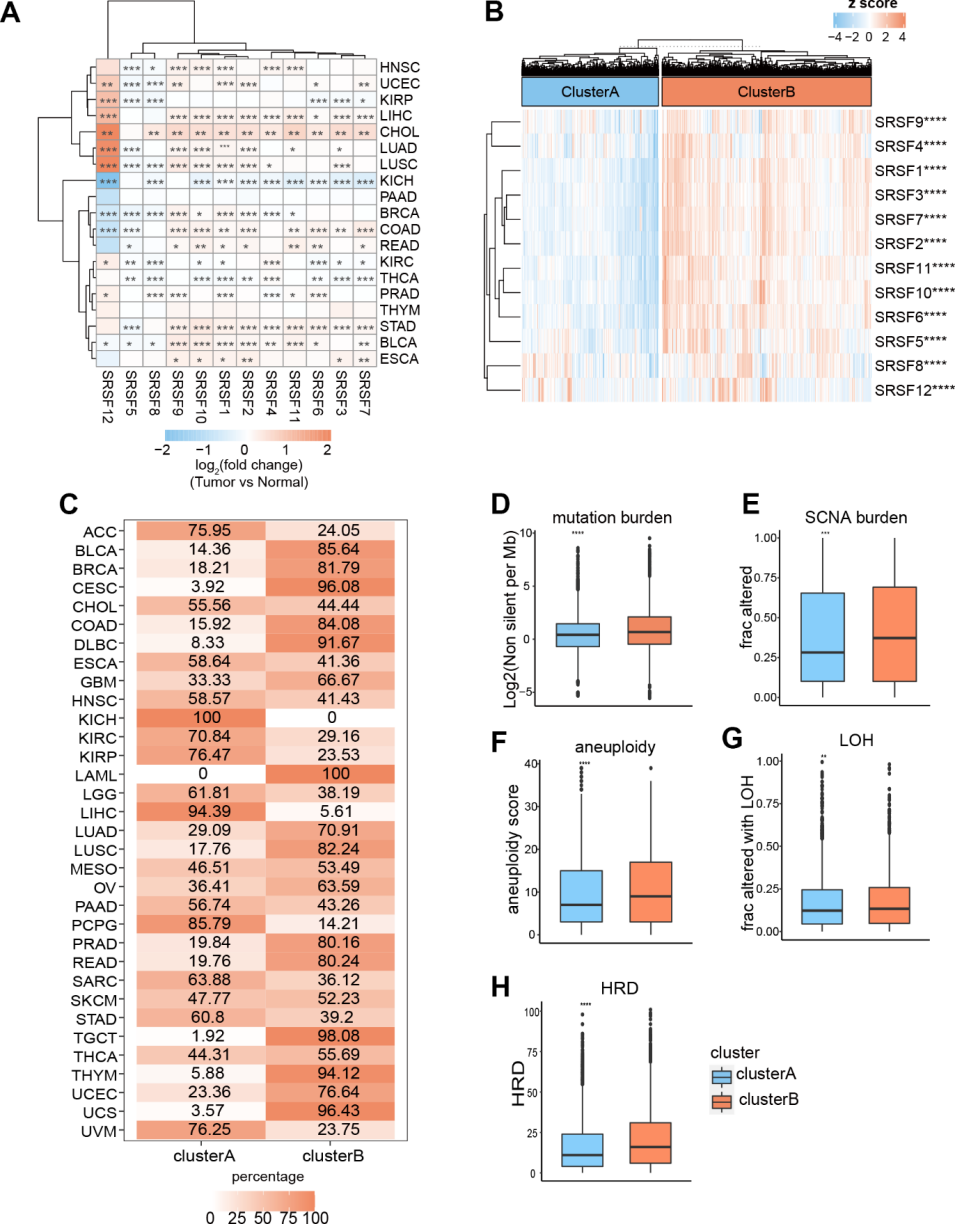
**Expression pattern of SRSFs and their association with genome instability in human cancer.** (**A**) Heatmap demonstrating differential expression of SRSFs between tumor and normal tissue. (**B**) Unsupervised consensus clustering based on SRSFs mRNA expression identifies two distinct patient clusters. (**C**) Sample distribution across 33 cancer types in the two patient clusters. The values represent the proportion of the two clusters in the different cancer types. (**D**–**H**) Differences in five scores (**D**, mutation burden; **E**, SCNA burden; **F**, aneuploidy scores; **G**, LOH; **H**, HRD) representing genome instability between the two clusters. The differences between the two clusters were tested by Wilcox’s rank sum test. The asterisks represented the statistical *P*-value (*P < 0.05; **P < 0.01; ***P < 0.001; ****P < 0.0001).

A thought-provoking question is whether these DNA alterations influence the expression of SRSFs. Thus, we investigated the expression perturbations of 12 SRSFs across 19 selected cancer types (Figure 3A). As expected, the SRSFs with CNV amplification displayed significantly higher expression in tumor tissues compared to normal ones (e.g., SRSF1, SRSF2, SRSF3, and SRSF6). In addition, we also noticed that despite CNV amplification or deep deletion in some SRSFs, there was no corresponding increase or decrease in mRNA expression level. For example, SRSF12 displayed CNV deep deletion in LIHC, LUAD, and PRAD; however, showing higher expression in cancer tissues. It is not unique, for example, the CNV alteration of SRSF10 in CHOL and its mRNA expression was also not synergistic (Figures 2D, 3A). We admit that gene expression is profoundly affected by CNV amplification and deletion [46]. Our findings suggested that specific to individual cancer types or genes, this relationship may not be fully established. We therefore believe that the CNV alterations are only one of the mechanisms, but not only, leading to expression perturbations of SRSFs. Taken together, the results demonstrate a picture of highly heterogeneous genetic and expression alteration of SRSFs across cancer types, suggesting that SRSFs dysregulation plays a significant role in different cancer contexts.

### Cluster analysis based on the pan-cancer SRSFs expression profiles identified two patient subgroups with significant genomic heterogeneity

To gain an insight into the integrated landscape of SRSFs expression, an unsupervised consensus clustering of all tumor samples from TCGA was performed, with the global expression pattern of SRSFs as the basis. Two distinct patient clusters were eventually identified, including 4079 cases in cluster A and 6248 cases in cluster B, with cluster B displaying a higher expression of all 12 SRSFs (Figure 3B). Further investigating the distribution of cancer types in the two clusters, we found that ACC, three hepatobiliary and pancreatic tumors (CHOL, LIHC and PAAD), two upper digestive tract tumors (ESCA, and STAD), HNSC, PCPG, and three kidney cancers (KICH, KIRC, KIRP) largely located in cluster A, whereas two lower digestive tract tumors (COAD, and READ), BLCA, BRCA, two lung cancers (LUAD and LUSC), three female reproductive system tumors (OV, UCEC, and UCS), TGCT, THYM and so on predominately resided in cluster B (Figure 3C). The results indicated there is a dysregulated expression profile of SRSFs in a cancer-type dependent manner.

Previous studies [47–50] have reported that SRSFs not only regulated RNA splicing, but also played important roles in transcriptional regulation. The co-transcriptional complex of SRSFs contributed to genomic stability, while abnormal expression of SRSFs might impair DNA stability [51]. So, we further assessed the genomic instability in the two clusters. Mutation and SCNA levels were used to characterize the genomic instability as previously described [17]. We used some scores, including mutation burden, SCNA burden, aneuploidy, LOH and HRD, to display the global mutations and SCNA levels [15]. We observed that cluster B, with higher expression of SRSFs, presented higher levels of all these scores reflecting genome instability (Figure 3D–3H). Genomic instability leads to abnormal expression of genes [52], which in turn contributes to genomic instability. Our finding indicated that this phenomenon might occur more in the cancer types of cluster B.

### DNA methylation, m6A modification and miRNAs regulate SRSFs expression in a cancer-type dependent manner

**Figure 4:**
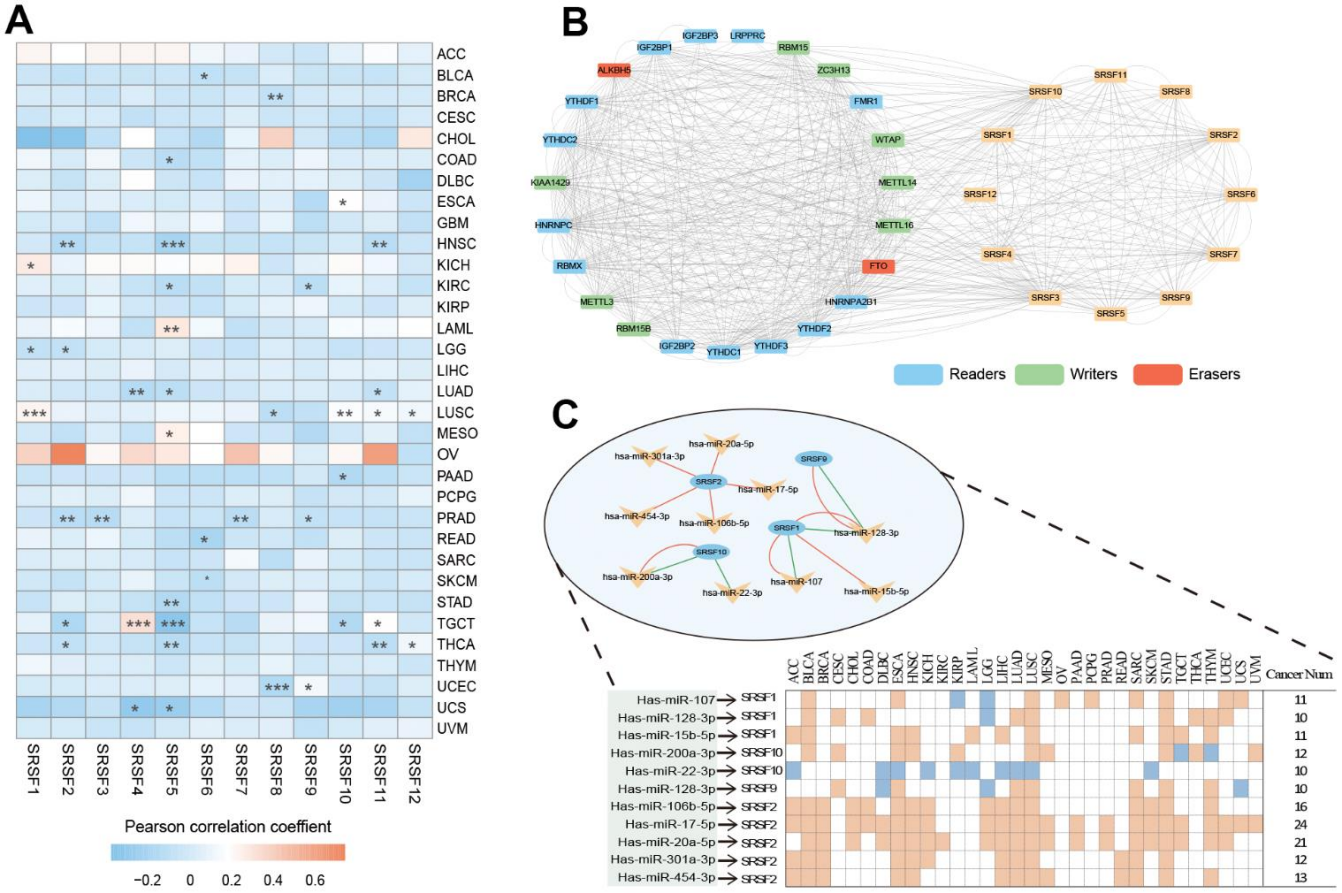
**DNA methylation, m6A modification and miRNA regulations of SRSFs.** (**A**) Heatmap demonstrating the correlation between DNA methylation level within promoters and SRSFs mRNA expression in each cancer type. (**B**) m6A-SRSFs protein-protein interaction network showing m6A regulators interacted with SRSFs frequently. (**C**) miRNA–SRSF interaction network and heatmap demonstrating miRNA-SRSF pairs displayed different correlation directions in different cancer contexts. Red represents a positive correlation, and green represents a negative correlation.

DNA methylation has long been recognized as an epigenetic determinant of gene expression [53–55], and DNA hypermethylation in promoter regions can lead to the downregulation of gene expression and gene silencing [56–58]. However, this relationship is not universal, at least in some special cases [59, 60]. An interesting question is worth exploring: how does DNA methylation affect the mRNA expression of SRSFs in human pan-cancer? To evaluate the relationships between SRSFs mRNA expression and DNA methylation levels, we investigated the correlation between 12 SRSFs expression and overall methylation levels in each cancer type. Our findings suggested that DNA methylation levels of SRSFs were negatively correlated with gene expression in most cancer types. However, when specific to certain cancer types or genes, this negative correlation did not hold, such as SRSF1 in LUSC and KICH; SRSF4 in TGCT, and SRSF5 in LAML and MESO (p < 0.05, Figure 4A).

m6A, as the most common internal modification of RNA, can affect various RNA biological processes [61, 62]. Previous studies [63–67] have reported that m6A regulators were extensively involved in RNA splicing process and interacted with splicing factors involved in the regulation of the splicing process, thereby affecting the expression level of many RNAs. To obtain a global picture of the association between m6A regulators and SRSFs in different cancer contexts, we performed a Pearson correlation analysis across 33 cancer types (Supplementary Table 1). Surprisingly, we noticed that in any cancer type, m6A regulators belonging to three different functional classifications (readers, writers, and erasers) presented highly correlated expression patterns with SRSFs, and this correlation was dominated by positive regulation. Moreover, we found that these 23 m6A regulators interacted with SRSFs frequently in protein-protein interaction networks (Figure 4B), which once again confirmed the close association of m6A modification with the expression of SRSFs. Our study further extended the perception of m6A-SRSF pairs. For example, a previous study [68] has confirmed that FTO controls mRNA splicing by modulating the RNA-binding capacity of SRSF2. However, the direction of this regulation (positive or negative) has not been established in a wide range of cancer types. Our findings showed that FTO was somewhat linked, either positively or negatively associated with SRSF2 in 19 cancer types. The same m6A-SRSF pair displayed completely opposite regulation directions in different cancer types, suggesting that m6A modification of SRSFs was cancer-type-dependent.

Massive reports [69–71] have demonstrated that miRNA was involved in gene expression regulation. Previous studies [72, 73] have piecemeally reported expression regulation patterns between miRNAs and SRSFs. To gain a more comprehensive understanding of the miRNA regulatory network of SRSFs, we identified all miRNAs with the potential to bind to SRSFs through strict screening criteria. From the overall distribution, we found that the same miRNA-SRSF pair had completely opposite regulation directions in different cancer types. For example, miR-101-3p and SRSF10 were negatively correlated in ACC, DLBC, LGG, LIHC and STAD, while they showed positive correlations in COAD, HNSC, KIRC, LUSC, OV, PAAD, TGCT, and THCA (Supplementary Table 2). Moreover, as we expected, the same miRNA could act with multiple different SRSFs, while different miRNAs could also act on the same SRSF. This phenomenon also corroborated the findings of Mahony et al. [74]. Through more stringent criteria (described in the Materials and Methods section) for further filtering miRNAs targeting SRSFs, we finally identified 11 miRNA-SRSF pairs (Figure 4C). These miRNA-SRSF pairs also displayed different correlation strengths and directions in different cancer context (Supplementary Table 3). For example, the miR-107-SRSF1 pair showed positive correlations in female reproductive system cancers (OV, UCEC and UCS) and upper digestive tract cancers (ESCA and STAD), while presenting negative correlation in KIRP and LGG (Figure 4C). Taken together, these results indicated that miRNA could regulate the expression of SRSFs, and whether this regulation was positive or negative depended on the cancer context.

### SRSFs associated oncogenic pathways

**Figure 5:**
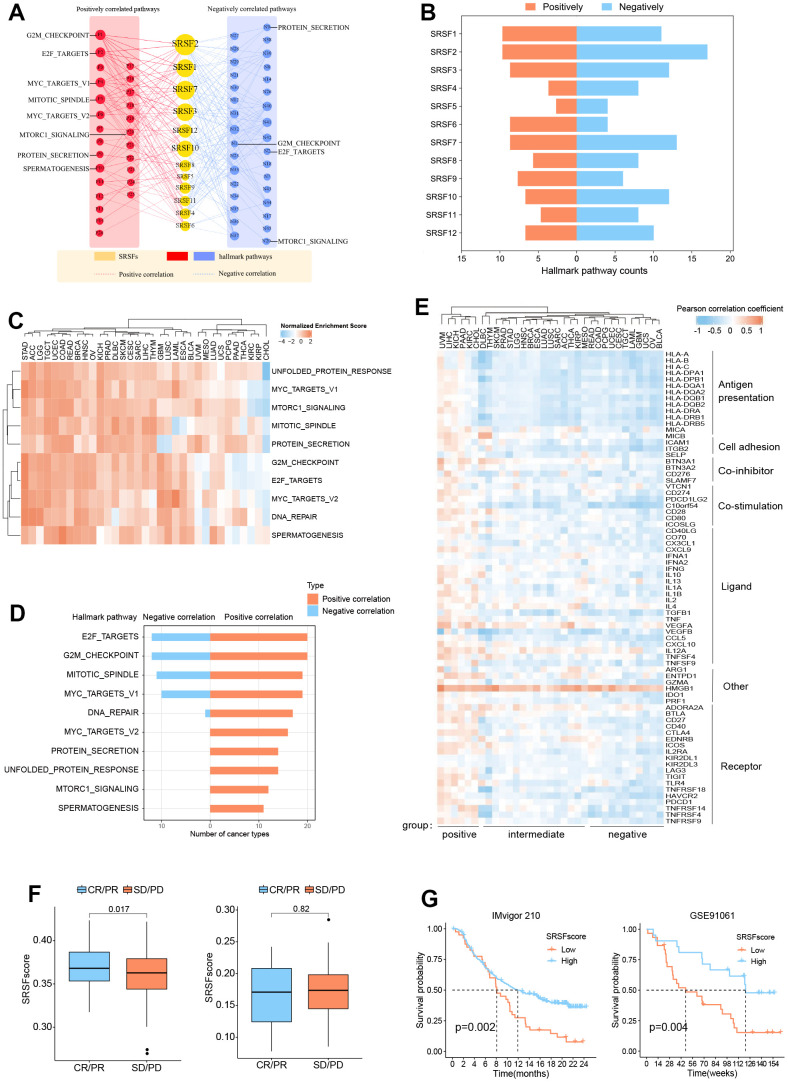
**SRSFs associated oncogenic pathways and immune links.** (**A**) SRSFs and hallmark pathways interaction network. Red and blue marks represent positive and negative correlation respectively. Node sizes correspond to the link numbers. (**B**) The count of hallmark pathways is related to individual SRSFs. (**C**) Heatmap demonstrating normalized enrichment score of significant pathways. We reported pathways that showed consistent significant correlations (q-value < 0.05) in at least 10 cancer types. (**D**) Barplots presenting number of cancer types which are negative and positive correlations between SRSFs and significant pathways. (**E**) Heatmap demonstrating Pearson correlation coefficient between SRSFscore and immunomodulator mRNA expression in each cancer type. Unsupervised clustering identified three subgroups with different correlations, including positive-, intermediate-, and negative-groups. (**F**) Differences in SRSFscore between the responder (CR/PR) and non-responder (SD/PD) groups. Left, in metastatic urothelial carcinoma; Right, in advanced melanoma. The differences between the two groups were tested by Wilcox’s rank sum test. (**G**) Kaplan-Meier survival curves showing the differences in overall survival between high and low SRSFscore patients. Left, in metastatic urothelial carcinoma; Right, in advanced melanoma. Statistical significance was assessed by log-rank test.

Given that previous studies [75, 76] have sporadically mentioned the involvement of SRSFs in cancer occurrence and progression, however lacking specific molecular mechanism to elucidate how SRSFs affect cancer from a global view, we investigated the relationship of individual SRSFs as well as overall SRSFs with the activity of 50 hallmark pathways across 33 cancer types. We noted that each SRSF was associated with multiple pathways, some with pathway activation and some with pathway inhibition (Figure 5A and Supplementary Table 4). The expressions of SRSF2, SRSF7, SRSF1 and SRSF3 were correlated to more activated pathways, such as the E2F targets, G2M checkpoint, MYC targets V1 and Hedgehog signalling. In particular, we observed that the SRSF2 was associated with most pathway activation (in 10/50 pathways) or inhibition (in 17/50 pathways), while SRSF5 was the opposite (Figure 5B). Our above findings suggested that DNA methylation, m6A modification and miRNA regulation of SRSFs were cancer-type-dependent. Therefore, we further explored the association of SRSFs expression with oncogenic pathway activity in different cancer types. As expected, we observed that whether SRSFs inhibited or activated the carcinogenic pathway was closely related to different cancer contexts (Supplementary Table 5). For example, SRSF2 expression was associated with pathway inhibition of DNA repair in MESO and SKCM, but the opposite was observed in LUAD, LUSC, as well as STAD. In addition, we also noted that SRSFs expression was correlated with the activation or inhibition of multiple immune-related pathways in different cancer types, such as interferon alpha response, interferon gamma response, Il-6/JAK/STST3 signaling and so on.

Previous studies [77, 78] have pointed out that genes functioned in concert, not in isolation. SRSFs, as the same gene family, share the same or similar functional properties. They may function more in a genetically synergistic manner. This was indirectly confirmed by SRSFs expression correlation analysis (Supplementary Figure 1) and protein-protein interaction network (Figure 4B). Therefore, we took SRSFs expression as a whole and further evaluated the relationship between overall SRSFs expression and oncogenic pathway activity. Here, “SRSFscore” was used to represent the estimated level of overall SRSFs expression as previously described [15, 25, 26]. From the results of GSEA analysis, we observed that through strict screening criteria, SRSFs had positive or negative correlation pathways in different cancer types (Figure 5C, 5D). We noted that E2F targets and G2M checkpoints were associated with SRSFs in 32 cancer types, with positive associations appearing in 20 cancer types and negative associations in 12 cancer types. This suggested that SRSFs played critical roles in regulating cell cycle and proliferation. Moreover, in different cancer types, the correlation between SRSFs and the same pathway also showed completely opposite directions (Figure 5D). For example, DNA repair was negatively correlated with SRSFs in LAML, but positively correlated with SRSFs in other cancer types (ACC, BLCA, BRCA, COAD, ESCA, GBM, HNSC, KICH, LGG, LIHC, LUSC, OV, PAAD, SARC, STAD, TGCT and UCEC), which again indicated that whether SRSFs inhibited or activated the carcinogenic pathway depended on cancer contexts.

### SRSFs are associated with cancer immunity

In recent years, the close relationship between cancer and immunity has gradually been deeply recognized [79–81]. Given the widespread genomic alterations and expression disturbances of SRSFs in various types of cancers and their close associations with multiple oncogenic pathways (especially immune-related pathways), we were interested in further investigating the potential links between SRSFs and cancer immunity. Xcell analysis revealed that SRSFs were significantly associated with the infiltration abundance of multiple immune or stromal cells in various cancer types (Supplementary Figure 2). We observed that CD8 ^+^ T cell infiltration abundance was negatively correlated with SRSFs expression in OV and UCEC, but positively correlated in BRCA, CHOL, COAD, KICH, KIRC, LIHC, LUSC, PAAD, PRAD, THYM, and UVM. A similar situation was also seen in most other cells (e.g., myeloid dendritic cell, endothelial cell, eosinophil, cancer associated fibroblast, etc.). This indicated a large heterogeneous association pattern between SRSFs and immunity in different cancer types. Additionally, we also examined the correlation between SRSFscore and some immunomodulators that are crucial in immunotherapy [14]. Our results suggested that SRSFs were negatively correlated with some common immune checkpoints (LAG3, CD274, CTLA4, TIGIT, PDCD1) in UCS, THYM, OV, CESC and BLCA, while positively correlated in UVM, STAD, PAAD, MESO, LUAD, LIHC, KIRC, KICH, HNSC, CHOL and BRCA (Figure 5E). Based on the correlation pattern between SRSFscore and immunomodulators, we clustered cancer types into three subgroups with different correlations, including positive-, intermediate-, and negative- groups. Interestingly, three female reproductive system tumors (OV, UCS and UCEC) were in the negative group, while two renal carcinomas (KICH and KIRC) and three cancer types from the hepatobiliary and pancreatic system (LIHC, CHOL, PAAD) were in the positive group. Strikingly, HMGB1 was found consistently positively correlated with the SRSFscore in all cancer types (Figure 5E).

Given that we identified the close link between SRSFs and cancer immunity, we therefore sought to further explore the potential impact of SRSFs on cancer immunotherapy. We obtained pre-immunotherapy gene expression data and clinical information from two cancer immunotherapy cohorts [28, 29] with relatively large sample sizes. According to tumor responses to immunotherapy, we classified the patients into responder (CR/PR) and non-responder (SD/PD) groups, and compared the differences in SRSFscore between the two groups. We found that high SRSFscore was associated with better immunotherapy efficacy in metastatic urothelial carcinoma cohort. But in advanced melanoma, we did not observe this (Figure 5F). We believed that the predictive value of SRSFscore as a hint of immunotherapy response might be influenced by different cancer contexts, as described above. Additionally, whether SRSFscore could indicate the prognostic risk of patients receiving immunotherapy was also worth exploring. Our results showed that high SRSFscore was associated with better prognosis in the two immunotherapy cohorts (Figure 5G), which further suggested the close relationship between SRSFs and cancer immunity.

### SRSFs displays the potential to predict survival

**Figure 6:**
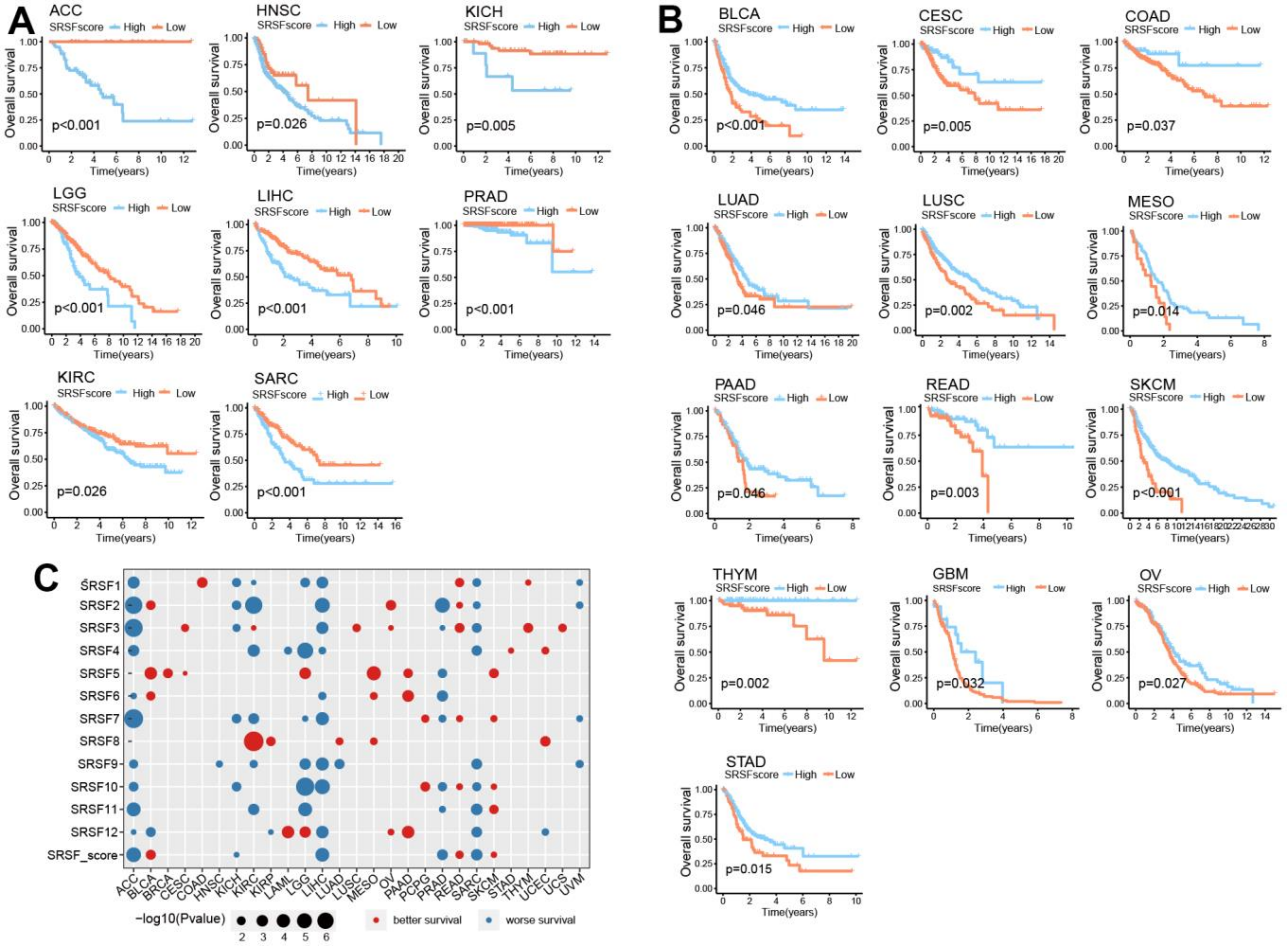
**SRSFscore displayed the potential to predict survival in 21 cancer types.** (**A**) Kaplan-Meier survival curves showing high SRSFscore is associated with worse survival in eight cancer types. (**B**) Kaplan-Meier survival curves showing high SRSFscore is related to better survival in thirteen cancer types. Differences in survival between two groups were tested using the log-rank test. (**C**) Bubble diagram showing the Cox regression correlation of SRSFs and survival. The diagram only displayed significant dots with *P*-value <0.05.

Above investigations identified the close association of SRSFs to cancer, so it was necessary to further determine the relationship between SRSFs and the survival of cancer patients. According to SRSFs expression level determined by rank statistics selected maximally, patients were divided into two groups to achieve a predictive value of SRSFs in survival [15]. Differences in survival between two groups were tested using the log-rank test. We observed that SRSFscore was significantly linked to patient survival in 21 cancer types (Figure 6A, 6B). Moreover, high SRSFscore could also indicate better survival in both metastatic urothelial carcinoma and advanced melanoma (Figure 5G). In ACC, HNSC, KICH, KIRC, LGG, LIHC, PRAD and SARC, high SRSFscore was associated with poor prognosis (Figure 6A), while SRSFscore was considered as a favorable prognostic factor in BLCA, CESC, COAD, LUAD, LUSC, MESO, PAAD, READ, SKCM, THYM, GBM, OV and STAD (Figure 6B). We also performed survival analysis on nine independent data cohorts from Gene Expression Omnibus involving five cancer types. The results again confirmed that high SRSFscore was associated with better survival of OV, LUAD, STAD and COAD, suggesting that SRSFs might be a key protective factor for OV, LUAD, STAD and COAD. Conversely, high SRSFscore was associated with poor prognosis of LIHC, suggesting that SRSF might be a risk factor in LIHC (Supplementary Figure 3).

In order to further investigated the contribution of each SRSF to the survival prediction value of SRSFscore, Cox regression analysis was performed on all 12 SRSFs and combined SRSFscore (Figure 6C). We observed that in ACC, KICH, LIHC, PRAD, and SARC, most SRSFs and SRSFscore showed consistent tendency toward worse survival. In the same cancer type, these 12 SRSFs did not all display direction-consistent prognostic risk. For example, in KIRC, SRSF1, SRSF2, SRSF4, SRSF7, SRSF9, SRSF11 were prognostic risk factors, while SRSF3 and SRSF8 were prognostic friendly factors. Similar cases were also seen in BLAC, KIRP, LAML, LGG, LUAD, and UCEC. In particular, for ACC, 11 of 12 SRSFs and SRSFscore; For LIHC, 10 of 12 SRSFs and SRSFscore; For SARC, 9 of 12 SRSFs and SRSFscore were all predictive of worse survival. Collectively, these findings suggested that SRSFs or SRSFscore had the potential to predict survival in certain cancer contexts.

### CellMiner analysis reveals the association of SRSFs to drug sensitivity

**Figure 7:**
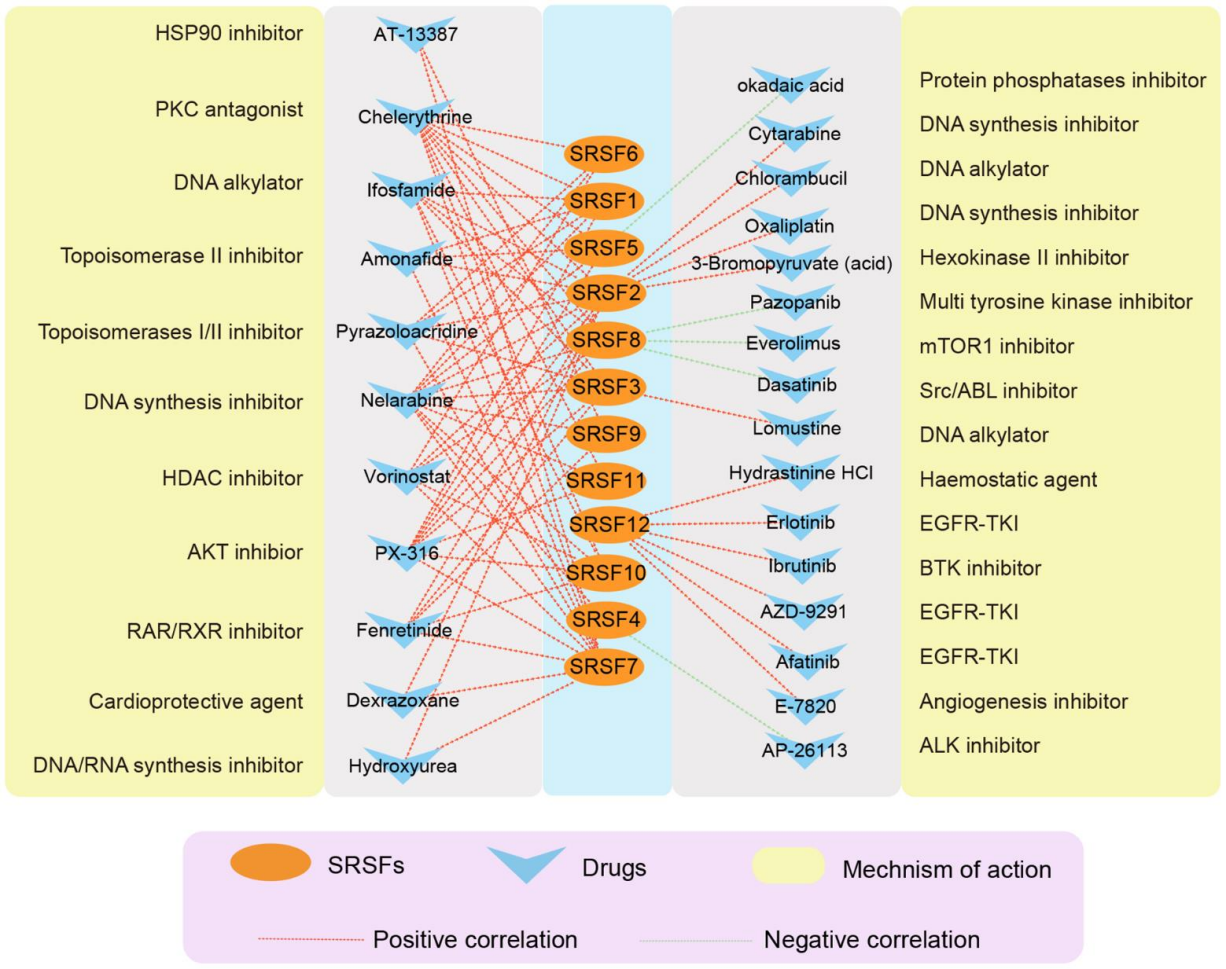
**The correlation network showing the association of SRSFs to drug sensitivity.** Red represents a positive correlation, and green represents a negative correlation.

A growing number of studies [82–84] have suggested that genomic and expression alterations played essential roles in drug responsiveness. Our study observed widespread genomic alterations and expression perturbations of SRSFs across cancer types. To further investigate the role of SRSFs on chemotherapy or targeted therapy, we performed drug sensitivity analysis, and calculated the Pearson correlation coefficients between Z scores of clinical trials and FDA-approved drugs and mRNA expression values for each SRSF. Through stringent screening criteria (|Pearson correlation coefficient| ≥ 0.3 and *P*-value < 0.01), we found 27 compounds, of which the sensitivity of 5 drugs (Dasatinib, Everolimus, Pazopanib, AP-26113 and Okadaic acid) was negatively correlated with gene expression and 22 drugs were positively correlated (Figure 7). Among these drugs, we found that 4 DNA synthesis inhibitors (Oxaliplatin, Nelarabine, Cytarabine and Hydroxyurea) were positively correlated with SRSFs expression, among which Nelarabine was positively correlated with 11 individual SRSFs, suggesting that the high expression of SRSFs might inhibit the sensitivity of tumor cells to DNA synthesis inhibitors. In contrast, overexpression of SRSFs (SRSF5 and SRSF8) might enhance the sensitivity of tumor to Src/ABL, mTOR1, and protein phosphatase inhibitors. These results suggested that the abnormal expression of SRSFs might mediate the resistance of tumor tissues to chemotherapy or targeted drug therapy.

## DISCUSSION

A growing number of studies [11, 12, 75, 76] have reported the important roles of SRSFs in tumorigenesis and progression. However, existing research on SRSFs mainly focus on single SRSFs or individual tumor types, which raises some interesting questions remained unsolved. For example, do SRSFs share common regulatory targets or molecular alteration characteristics in different cancer contexts? Is the regulatory mechanism similar for all these targets? Do diverse SRSFs share similar oncogenic pathways within the same cancer type? To clarify these doubts, it is particularly important to depict a global landscape of SRSFs in pan-cancer from multiple perspectives.

In this study, we performed a systematic characterization of SRSFs in over 10000 samples across 33 cancer types. Our results not only revealed diverse molecular characteristics and potential mechanisms of SRSFs in different cancer contexts, but also uncovered common SRSFs related oncogenic pathways, thus depicting a global landscape of SRSFs regulation in human pan-cancer.

In the genomics analysis, we observed widespread genomic alterations of SRSFs in multiple cancer types. SCNA, especially CNV amplification, accounted for a very large proportion of DNA alterations. Thus, we speculated that at the genomic level, it was SCNA, especially CNV amplification, but not mutation, which was the major cause of SRSFs dysregulation in cancer. Unlike the previous description by Luo et al. [15] that gene CNV was significantly associated with expression, we found that not all SRSFs expression changed with CNV amplification or deletion. For example, SRSF12 presented CNV deep deletion in LIHC, LUAD, and PRAD, however showing higher expression in cancer tissues. The CNV alteration of SRSF10 in CHOL and its mRNA expression was also not synergistic. Previous studies [85–87] have reported that SRSFs expression was dysregulated in a variety of cancers, which was consistent with our findings. Abnormal expression of SRSFs usually lead to changes in cancer-related pathways (e.g., epithelial mesenchymal transformation) [88] and alternative splicing [89]. Additionally, we found that SRSFs expression was closely related to the activation or inhibition of various carcinogenic pathways, such as E2F target, G2M checkpoint, DNA repair, mTORC1 signaling, etc. However, same SRSFs presented completely opposite regulatory directions with the same oncogenic pathway in different cancer types, suggesting that the effect of SRSFs on the specific pathway was dependent on cancer contexts. Similar findings were observed in Luo et al. [15]. Overall, SRSFs experienced widespread genomic alterations and expression dysregulation in multiple cancers, which in turn could affect key oncogenic pathways and contribute to tumorigenesis and progression [90, 91].

In pathway analysis and cancer immune correlation analysis, we observed that SRSFs were associated with the activation or inhibition of multiple immune pathways, and were also closely related to the expression of multiple immune checkpoints (LAG3, CD274, CTLA4, TIGIT, PDCD1). Interestingly, we found that SRSFs displayed negative associations with more immunoregulators in female reproductive system tumors (OV, UCS and UCEC), while positive in two renal tumors (KICH and KIRC) and tumors derived from the hepatobiliary-pancreatic system (LIHC, CHOL, PAAD). This study is the first to discover this interesting phenomenon, which will provide a new perspective for the in-depth study of SRSFs in cancer immunotherapy.

SRSFs experienced widespread genomic alterations and dysregulated expression in various types of cancers, and were closely related to multiple immune pathways and immunomodulators. This may provide important insights for the development of clinical translational medicine. A previous study [92] has reported that in leukemia, mutant SRSF2 cells were more sensitive to E71079, a splicing-altering compound that binds to the SF3B complex, than wild-type SRSF2 cells [93, 94]. So, we believe that SRSFs may be key biomarkers for predicting cancer treatment response in the near future. Our study found that in metastatic urothelial carcinoma, SRSFs could predict response to PD-1 blockade therapy, whereas in advanced melanoma, this predictive value was not accepted. We believed that SRSFs predicted response to immunotherapy depending on cancer contexts. In the following survival analysis, we found that both in metastatic urological carcinoma and in advanced melanoma, SRSFs could well predict patient prognosis, that is, higher SRSFscore was associated with better survival. The potential mechanisms underlying the different predictive values of SRSFs in specific cancer types remained poorly defined. In the future, individualized development of immunotherapy still requires a lot of work to understand its detailed mechanisms.

Chemical resistance is responsible for the failure of cancer treatment. Splicing disorders play a critical role in tumorigenesis [95], and the involvement of SRSFs in cancer resistance to chemotherapy remains elusive [96]. It has been reported that SRSF4 played important roles in the process of cisplatin affecting splicing, and that reduced SRSF4 expression inhibited multiple cisplatin-induced splicing alterations and cell death [97]. Fukuhara et al. [98] suggested that targeting kinases involved in alternative splicing by regulating the phosphorylation of SRSFs was a good option for splicing regulation. SRSFs could be phosphorylated by their kinases (SRPK1 and SRPK2), as well as by DNA topoisomerase. In addition, Pilch et al. [99] reported that NB-506, acting as a topoisomerase inhibitor, could regulate the phosphorylation and splicing patterns of SRSF1 and thereby regulate gene expression. Consistent with this, our study also found that the sensitivity of two DNA topoisomerase inhibitors (Amonafide and Pyrazoloacridine) were positively correlated with SRSFs expression. In addition, we observed that the sensitivity of other chemotherapeutic drugs (e.g., DNA synthesis inhibitors) and targeted drugs (e.g., EGFR-TKI) were also associated with the expression of SRSFs. Therefore, we believe that targeting SRSFs may be a cancer therapy approach with great potential for clinical application in the future. The effects of these drugs on SRSFs expression and tumor growth, as well as the specific mechanisms by which SRSFs are involved in tumor resistance, are still worthy of further investigation.

Importantly, we noted that the regulations associated with SRSFs were all cancer context-dependent, including genomic alterations, dysregulated expression, DNA methylation, m6A modification, miRNA, oncogenic pathways, etc. This property also determined the varying predictive potentials of SRSFs for drug response and survival in different cancer types. These findings substantiated once again the nature of intertumor diversity [100, 101], and highlighted the importance of individualized cancer treatment.

To sum up, we systematically characterized the molecular characteristics and clinical relevance of SRSFs from multiple levels in over 10000 patients across 33 cancer types. However, in this study, a systematic characterization of SRSFs was performed using pan-cancer bulk data, but wet experiments are lacking. We will try to clarify the oncogenic patterns and mechanisms of specific SRSFs molecules in future studies and explore their potential anti-cancer strategies. The current study highlights the pivotal roles of SRSFs in cancer progression, provides rich resources for understanding the biological characteristics of SRSFs, which will contribute to the development of therapeutic strategies based on RNA alternative splicing.

## Supplementary Material

Supplementary Figures

Supplementary Table 1

Supplementary Table 2

Supplementary Table 3

Supplementary Table 4

Supplementary Table 5

## References

[1] Crews LA, Balaian L, Delos Santos NP, Leu HS, Court AC, Lazzari E, Sadarangani A, Zipeto MA, La Clair JJ, Villa R, Kulidjian A, Storb R, Morris SR, et al. RNA Splicing Modulation Selectively Impairs Leukemia Stem Cell Maintenance in Secondary Human AML. Cell Stem Cell. 2016; 19:599–612. 10.1016/j.stem.2016.08.003 27570067 PMC5097015

[2] Magomedova L, Tiefenbach J, Zilberman E, Le Billan F, Voisin V, Saikali M, Boivin V, Robitaille M, Gueroussov S, Irimia M, Ray D, Patel R, Xu C, et al. ARGLU1 is a transcriptional coactivator and splicing regulator important for stress hormone signaling and development. Nucleic Acids Res. 2019; 47:2856–70. 10.1093/nar/gkz010 30698747 PMC6451108

[3] Scotti MM, Swanson MS. RNA mis-splicing in disease. Nat Rev Genet. 2016; 17:19–32. 10.1038/nrg.2015.3 26593421 PMC5993438

[4] Karni R, de Stanchina E, Lowe SW, Sinha R, Mu D, Krainer AR. The gene encoding the splicing factor SF2/ASF is a proto-oncogene. Nat Struct Mol Biol. 2007; 14:185–93. 10.1038/nsmb1209 17310252 PMC4595851

[5] Oltean S, Bates DO. Hallmarks of alternative splicing in cancer. Oncogene. 2014; 33:5311–8. 10.1038/onc.2013.533 24336324

[6] Mogilevsky M, Shimshon O, Kumar S, Mogilevsky A, Keshet E, Yavin E, Heyd F, Karni R. Modulation of MKNK2 alternative splicing by splice-switching oligonucleotides as a novel approach for glioblastoma treatment. Nucleic Acids Res. 2018; 46:11396–404. 10.1093/nar/gky921 30329087 PMC6265459

[7] Erkelenz S, Mueller WF, Evans MS, Busch A, Schöneweis K, Hertel KJ, Schaal H. Position-dependent splicing activation and repression by SR and hnRNP proteins rely on common mechanisms. RNA. 2013; 19:96–102. 10.1261/rna.037044.112 23175589 PMC3527730

[8] Radzisheuskaya A, Shliaha PV, Grinev V, Lorenzini E, Kovalchuk S, Shlyueva D, Gorshkov V, Hendrickson RC, Jensen ON, Helin K. PRMT5 methylome profiling uncovers a direct link to splicing regulation in acute myeloid leukemia. Nat Struct Mol Biol. 2019; 26:999–1012. 10.1038/s41594-019-0313-z 31611688 PMC6858565

[9] Yoshimi A, Abdel-Wahab O. Molecular Pathways: Understanding and Targeting Mutant Spliceosomal Proteins. Clin Cancer Res. 2017; 23:336–41. 10.1158/1078-0432.CCR-16-0131 27836865 PMC5241248

[10] Braun S, Enculescu M, Setty ST, Cortés-López M, de Almeida BP, Sutandy FX, Schulz L, Busch A, Seiler M, Ebersberger S, Barbosa-Morais NL, Legewie S, König J, Zarnack K. Decoding a cancer-relevant splicing decision in the RON proto-oncogene using high-throughput mutagenesis. Nat Commun. 2018; 9:3315. 10.1038/s41467-018-05748-7 30120239 PMC6098099

[11] Pandit S, Zhou Y, Shiue L, Coutinho-Mansfield G, Li H, Qiu J, Huang J, Yeo GW, Ares M Jr, Fu XD. Genome-wide analysis reveals SR protein cooperation and competition in regulated splicing. Mol Cell. 2013; 50:223–35. 10.1016/j.molcel.2013.03.001 23562324 PMC3640356

[12] Savisaar R, Hurst LD. Both Maintenance and Avoidance of RNA-Binding Protein Interactions Constrain Coding Sequence Evolution. Mol Biol Evol. 2017; 34:1110–26. 10.1093/molbev/msx061 28138077 PMC5400389

[13] Zhang Y, Kwok-Shing Ng P, Kucherlapati M, Chen F, Liu Y, Tsang YH, de Velasco G, Jeong KJ, Akbani R, Hadjipanayis A, Pantazi A, Bristow CA, Lee E, et al. A Pan-Cancer Proteogenomic Atlas of PI3K/AKT/mTOR Pathway Alterations. Cancer Cell. 2017; 31:820–32.e3. 10.1016/j.ccell.2017.04.013 28528867 PMC5502825

[14] Thorsson V, Gibbs DL, Brown SD, Wolf D, Bortone DS, Ou Yang TH, Porta-Pardo E, Gao GF, Plaisier CL, Eddy JA, Ziv E, Culhane AC, Paull EO, et al, and Cancer Genome Atlas Research Network. The Immune Landscape of Cancer. Immunity. 2018; 48:812–30.e14. 10.1016/j.immuni.2018.03.023 29628290 PMC5982584

[15] Luo Z, Liu W, Sun P, Wang F, Feng X. Pan-cancer analyses reveal regulation and clinical outcome association of the shelterin complex in cancer. Brief Bioinform. 2021; 22:bbaa441. 10.1093/bib/bbaa441 33497432

[16] Mermel CH, Schumacher SE, Hill B, Meyerson ML, Beroukhim R, Getz G. GISTIC2.0 facilitates sensitive and confident localization of the targets of focal somatic copy-number alteration in human cancers. Genome Biol. 2011; 12:R41. 10.1186/gb-2011-12-4-r41 21527027 PMC3218867

[17] Knijnenburg TA, Wang L, Zimmermann MT, Chambwe N, Gao GF, Cherniack AD, Fan H, Shen H, Way GP, Greene CS, Liu Y, Akbani R, Feng B, et al, and Cancer Genome Atlas Research Network. Genomic and Molecular Landscape of DNA Damage Repair Deficiency across The Cancer Genome Atlas. Cell Rep. 2018; 23:239–54.e6. 10.1016/j.celrep.2018.03.076 29617664 PMC5961503

[18] Wilkerson MD, Hayes DN. ConsensusClusterPlus: a class discovery tool with confidence assessments and item tracking. Bioinformatics. 2010; 26:1572–3. 10.1093/bioinformatics/btq170 20427518 PMC2881355

[19] Chen XY, Zhang J, Zhu JS. The role of m^6^A RNA methylation in human cancer. Mol Cancer. 2019; 18:103. 10.1186/s12943-019-1033-z 31142332 PMC6540575

[20] Warda AS, Kretschmer J, Hackert P, Lenz C, Urlaub H, Höbartner C, Sloan KE, Bohnsack MT. Human METTL16 is a *N*^6^-methyladenosine (m^6^A) methyltransferase that targets pre-mRNAs and various non-coding RNAs. EMBO Rep. 2017; 18:2004–14. 10.15252/embr.201744940 29051200 PMC5666602

[21] Li Y, Gu J, Xu F, Zhu Q, Chen Y, Ge D, Lu C. Molecular characterization, biological function, tumor microenvironment association and clinical significance of m6A regulators in lung adenocarcinoma. Brief Bioinform. 2021; 22:bbaa225. 10.1093/bib/bbaa225 33003204

[22] Yang Y, Hsu PJ, Chen YS, Yang YG. Dynamic transcriptomic m^6^A decoration: writers, erasers, readers and functions in RNA metabolism. Cell Res. 2018; 28:616–24. 10.1038/s41422-018-0040-8 29789545 PMC5993786

[23] Hart T, Komori HK, LaMere S, Podshivalova K, Salomon DR. Finding the active genes in deep RNA-seq gene expression studies. BMC Genomics. 2013; 14:778. 10.1186/1471-2164-14-778 24215113 PMC3870982

[24] Hänzelmann S, Castelo R, Guinney J. GSVA: gene set variation analysis for microarray and RNA-seq data. BMC Bioinformatics. 2013; 14:7. 10.1186/1471-2105-14-7 23323831 PMC3618321

[25] Wang Y, Xu X, Maglic D, Dill MT, Mojumdar K, Ng PK, Jeong KJ, Tsang YH, Moreno D, Bhavana VH, Peng X, Ge Z, Chen H, et al, and Cancer Genome Atlas Research Network. Comprehensive Molecular Characterization of the Hippo Signaling Pathway in Cancer. Cell Rep. 2018; 25:1304–17.e5. 10.1016/j.celrep.2018.10.001 30380420 PMC6326181

[26] Korkut A, Zaidi S, Kanchi RS, Rao S, Gough NR, Schultz A, Li X, Lorenzi PL, Berger AC, Robertson G, Kwong LN, Datto M, Roszik J, et al, and Cancer Genome Atlas Research Network. A Pan-Cancer Analysis Reveals High-Frequency Genetic Alterations in Mediators of Signaling by the TGF-β Superfamily. Cell Syst. 2018; 7:422–37.e7. 10.1016/j.cels.2018.08.010 30268436 PMC6370347

[27] Aran D, Hu Z, Butte AJ. xCell: digitally portraying the tissue cellular heterogeneity landscape. Genome Biol. 2017; 18:220. 10.1186/s13059-017-1349-1 29141660 PMC5688663

[28] Mariathasan S, Turley SJ, Nickles D, Castiglioni A, Yuen K, Wang Y, Kadel EE II, Koeppen H, Astarita JL, Cubas R, Jhunjhunwala S, Banchereau R, Yang Y, et al. TGFβ attenuates tumour response to PD-L1 blockade by contributing to exclusion of T cells. Nature. 2018; 554:544–8. 10.1038/nature25501 29443960 PMC6028240

[29] Riaz N, Havel JJ, Makarov V, Desrichard A, Urba WJ, Sims JS, Hodi FS, Martín-Algarra S, Mandal R, Sharfman WH, Bhatia S, Hwu WJ, Gajewski TF, et al. Tumor and Microenvironment Evolution during Immunotherapy with Nivolumab. Cell. 2017; 171:934–49.e16. 10.1016/j.cell.2017.09.028 29033130 PMC5685550

[30] Roessler S, Jia HL, Budhu A, Forgues M, Ye QH, Lee JS, Thorgeirsson SS, Sun Z, Tang ZY, Qin LX, Wang XW. A unique metastasis gene signature enables prediction of tumor relapse in early-stage hepatocellular carcinoma patients. Cancer Res. 2010; 70:10202–12. 10.1158/0008-5472.CAN-10-2607 21159642 PMC3064515

[31] Zhao X, Parpart S, Takai A, Roessler S, Budhu A, Yu Z, Blank M, Zhang YE, Jia HL, Ye QH, Qin LX, Tang ZY, Thorgeirsson SS, Wang XW. Integrative genomics identifies YY1AP1 as an oncogenic driver in EpCAM(+) AFP(+) hepatocellular carcinoma. Oncogene. 2015; 34:5095–104. 10.1038/onc.2014.438 25597408 PMC4506915

[32] Muratani M, Deng N, Ooi WF, Lin SJ, Xing M, Xu C, Qamra A, Tay ST, Malik S, Wu J, Lee MH, Zhang S, Tan LL, et al. Nanoscale chromatin profiling of gastric adenocarcinoma reveals cancer-associated cryptic promoters and somatically acquired regulatory elements. Nat Commun. 2014; 5:4361. 10.1038/ncomms5361 25008978

[33] Ooi CH, Ivanova T, Wu J, Lee M, Tan IB, Tao J, Ward L, Koo JH, Gopalakrishnan V, Zhu Y, Cheng LL, Lee J, Rha SY, et al. Oncogenic pathway combinations predict clinical prognosis in gastric cancer. PLoS Genet. 2009; 5:e1000676. 10.1371/journal.pgen.1000676 19798449 PMC2748685

[34] Chia NY, Deng N, Das K, Huang D, Hu L, Zhu Y, Lim KH, Lee MH, Wu J, Sam XX, Tan GS, Wan WK, Yu W, et al. Regulatory crosstalk between lineage-survival oncogenes KLF5, GATA4 and GATA6 cooperatively promotes gastric cancer development. Gut. 2015; 64:707–19. 10.1136/gutjnl-2013-306596 25053715

[35] Marchion DC, Cottrill HM, Xiong Y, Chen N, Bicaku E, Fulp WJ, Bansal N, Chon HS, Stickles XB, Kamath SG, Hakam A, Li L, Su D, et al. BAD phosphorylation determines ovarian cancer chemosensitivity and patient survival. Clin Cancer Res. 2011; 17:6356–66. 10.1158/1078-0432.CCR-11-0735 21849418 PMC3186862

[36] Rousseaux S, Debernardi A, Jacquiau B, Vitte AL, Vesin A, Nagy-Mignotte H, Moro-Sibilot D, Brichon PY, Lantuejoul S, Hainaut P, Laffaire J, de Reyniès A, Beer DG, et al. Ectopic activation of germline and placental genes identifies aggressive metastasis-prone lung cancers. Sci Transl Med. 2013; 5:186ra66. 10.1126/scitranslmed.3005723 23698379 PMC4818008

[37] Okayama H, Kohno T, Ishii Y, Shimada Y, Shiraishi K, Iwakawa R, Furuta K, Tsuta K, Shibata T, Yamamoto S, Watanabe S, Sakamoto H, Kumamoto K, et al. Identification of genes upregulated in ALK-positive and EGFR/KRAS/ALK-negative lung adenocarcinomas. Cancer Res. 2012; 72:100–11. 10.1158/0008-5472.CAN-11-1403 22080568

[38] Yamauchi M, Yamaguchi R, Nakata A, Kohno T, Nagasaki M, Shimamura T, Imoto S, Saito A, Ueno K, Hatanaka Y, Yoshida R, Higuchi T, Nomura M, et al. Epidermal growth factor receptor tyrosine kinase defines critical prognostic genes of stage I lung adenocarcinoma. PLoS One. 2012; 7:e43923. 10.1371/journal.pone.0043923 23028479 PMC3446964

[39] Marisa L, de Reyniès A, Duval A, Selves J, Gaub MP, Vescovo L, Etienne-Grimaldi MC, Schiappa R, Guenot D, Ayadi M, Kirzin S, Chazal M, Fléjou JF, et al. Gene expression classification of colon cancer into molecular subtypes: characterization, validation, and prognostic value. PLoS Med. 2013; 10:e1001453. 10.1371/journal.pmed.1001453 23700391 PMC3660251

[40] Der SD, Sykes J, Pintilie M, Zhu CQ, Strumpf D, Liu N, Jurisica I, Shepherd FA, Tsao MS. Validation of a histology-independent prognostic gene signature for early-stage, non-small-cell lung cancer including stage IA patients. J Thorac Oncol. 2014; 9:59–64. 10.1097/JTO.0000000000000042 24305008

[41] Qian Z, Zhu G, Tang L, Wang M, Zhang L, Fu J, Huang C, Fan S, Sun Y, Lv J, Dong H, Gao B, Su X, et al. Whole genome gene copy number profiling of gastric cancer identifies PAK1 and KRAS gene amplification as therapy targets. Genes Chromosomes Cancer. 2014; 53:883–94. 10.1002/gcc.22196 24935174

[42] Schabath MB, Welsh EA, Fulp WJ, Chen L, Teer JK, Thompson ZJ, Engel BE, Xie M, Berglund AE, Creelan BC, Antonia SJ, Gray JE, Eschrich SA, et al. Differential association of STK11 and TP53 with KRAS mutation-associated gene expression, proliferation and immune surveillance in lung adenocarcinoma. Oncogene. 2016; 35:3209–16. 10.1038/onc.2015.375 26477306 PMC4837098

[43] Reinhold WC, Sunshine M, Liu H, Varma S, Kohn KW, Morris J, Doroshow J, Pommier Y. CellMiner: a web-based suite of genomic and pharmacologic tools to explore transcript and drug patterns in the NCI-60 cell line set. Cancer Res. 2012; 72:3499–511. 10.1158/0008-5472.CAN-12-1370 22802077 PMC3399763

[44] Bailey MH, Tokheim C, Porta-Pardo E, Sengupta S, Bertrand D, Weerasinghe A, Colaprico A, Wendl MC, Kim J, Reardon B, Ng PK, Jeong KJ, Cao S, et al, MC3 Working Group, and Cancer Genome Atlas Research Network. Comprehensive Characterization of Cancer Driver Genes and Mutations. Cell. 2018; 173:371–85.e18. 10.1016/j.cell.2018.02.060 29625053 PMC6029450

[45] Li Y, Xiao J, Bai J, Tian Y, Qu Y, Chen X, Wang Q, Li X, Zhang Y, Xu J. Molecular characterization and clinical relevance of m^6^A regulators across 33 cancer types. Mol Cancer. 2019; 18:137. 10.1186/s12943-019-1066-3 31521193 PMC6744659

[46] Beroukhim R, Mermel CH, Porter D, Wei G, Raychaudhuri S, Donovan J, Barretina J, Boehm JS, Dobson J, Urashima M, Mc Henry KT, Pinchback RM, Ligon AH, et al. The landscape of somatic copy-number alteration across human cancers. Nature. 2010; 463:899–905. 10.1038/nature08822 20164920 PMC2826709

[47] Shen M, Mattox W. Activation and repression functions of an SR splicing regulator depend on exonic versus intronic-binding position. Nucleic Acids Res. 2012; 40:428–37. 10.1093/nar/gkr713 21914724 PMC3245930

[48] Misteli T, Cáceres JF, Spector DL. The dynamics of a pre-mRNA splicing factor in living cells. Nature. 1997; 387:523–7. 10.1038/387523a0 9168118

[49] Das R, Yu J, Zhang Z, Gygi MP, Krainer AR, Gygi SP, Reed R. SR proteins function in coupling RNAP II transcription to pre-mRNA splicing. Mol Cell. 2007; 26:867–81. 10.1016/j.molcel.2007.05.036 17588520

[50] Lin S, Coutinho-Mansfield G, Wang D, Pandit S, Fu XD. The splicing factor SC35 has an active role in transcriptional elongation. Nat Struct Mol Biol. 2008; 15:819–26. 10.1038/nsmb.1461 18641664 PMC2574591

[51] Li X, Manley JL. Inactivation of the SR protein splicing factor ASF/SF2 results in genomic instability. Cell. 2005; 122:365–78. 10.1016/j.cell.2005.06.008 16096057

[52] Negrini S, Gorgoulis VG, Halazonetis TD. Genomic instability--an evolving hallmark of cancer. Nat Rev Mol Cell Biol. 2010; 11:220–8. 10.1038/nrm2858 20177397

[53] Ai T, Zhang J, Wang X, Zheng X, Qin X, Zhang Q, Li W, Hu W, Lin J, Chen F. DNA methylation profile is associated with the osteogenic potential of three distinct human odontogenic stem cells. Signal Transduct Target Ther. 2018; 3:1. 10.1038/s41392-017-0001-6 29527327 PMC5837092

[54] Kawakatsu T, Nery JR, Castanon R, Ecker JR. Dynamic DNA methylation reconfiguration during seed development and germination. Genome Biol. 2017; 18:171. 10.1186/s13059-017-1251-x 28911331 PMC5599895

[55] Moro L, Simoneschi D, Kurz E, Arbini AA, Jang S, Guaragnella N, Giannattasio S, Wang W, Chen YA, Pires G, Dang A, Hernandez E, Kapur P, et al. Epigenetic silencing of the ubiquitin ligase subunit FBXL7 impairs c-SRC degradation and promotes epithelial-to-mesenchymal transition and metastasis. Nat Cell Biol. 2020; 22:1130–42. 10.1038/s41556-020-0560-6 32839549 PMC7484425

[56] Zhao JY, Liang L, Gu X, Li Z, Wu S, Sun L, Atianjoh FE, Feng J, Mo K, Jia S, Lutz BM, Bekker A, Nestler EJ, Tao YX. DNA methyltransferase DNMT3a contributes to neuropathic pain by repressing Kcna2 in primary afferent neurons. Nat Commun. 2017; 8:14712. 10.1038/ncomms14712 28270689 PMC5344974

[57] Restrepo A, Smith CA, Agnihotri S, Shekarforoush M, Kongkham PN, Seol HJ, Northcott P, Rutka JT. Epigenetic regulation of glial fibrillary acidic protein by DNA methylation in human malignant gliomas. Neuro Oncol. 2011; 13:42–50. 10.1093/neuonc/noq145 21075782 PMC3018916

[58] Xu J, Chen G, Hermanson PJ, Xu Q, Sun C, Chen W, Kan Q, Li M, Crisp PA, Yan J, Li L, Springer NM, Li Q. Population-level analysis reveals the widespread occurrence and phenotypic consequence of DNA methylation variation not tagged by genetic variation in maize. Genome Biol. 2019; 20:243. 10.1186/s13059-019-1859-0 31744513 PMC6862797

[59] Madsen A, Höppner G, Krause J, Hirt MN, Laufer SD, Schweizer M, Tan WL, Mosqueira D, Anene-Nzelu CG, Lim I, Foo RS, Hansen A, Eschenhagen T, Stenzig J. An Important Role for DNMT3A-Mediated DNA Methylation in Cardiomyocyte Metabolism and Contractility. Circulation. 2020; 142:1562–78. 10.1161/CIRCULATIONAHA.119.044444 32885664 PMC7566310

[60] Lee DD, Komosa M, Nunes NM, Tabori U. DNA methylation of the TERT promoter and its impact on human cancer. Curr Opin Genet Dev. 2020; 60:17–24. 10.1016/j.gde.2020.02.003 32114294

[61] Molinie B, Wang J, Lim KS, Hillebrand R, Lu ZX, Van Wittenberghe N, Howard BD, Daneshvar K, Mullen AC, Dedon P, Xing Y, Giallourakis CC. m(6)A-LAIC-seq reveals the census and complexity of the m(6)A epitranscriptome. Nat Methods. 2016; 13:692–8. 10.1038/nmeth.3898 27376769 PMC5704921

[62] Lu M, Zhang Z, Xue M, Zhao BS, Harder O, Li A, Liang X, Gao TZ, Xu Y, Zhou J, Feng Z, Niewiesk S, Peeples ME, He C, Li J. N^6^-methyladenosine modification enables viral RNA to escape recognition by RNA sensor RIG-I. Nat Microbiol. 2020; 5:584–98. 10.1038/s41564-019-0653-9 32015498 PMC7137398

[63] Liu F, Clark W, Luo G, Wang X, Fu Y, Wei J, Wang X, Hao Z, Dai Q, Zheng G, Ma H, Han D, Evans M, et al. ALKBH1-Mediated tRNA Demethylation Regulates Translation. Cell. 2016; 167:816–28.e16. 10.1016/j.cell.2016.09.038 27745969 PMC5119773

[64] Yan F, Al-Kali A, Zhang Z, Liu J, Pang J, Zhao N, He C, Litzow MR, Liu S. A dynamic N^6^-methyladenosine methylome regulates intrinsic and acquired resistance to tyrosine kinase inhibitors. Cell Res. 2018; 28:1062–76. 10.1038/s41422-018-0097-4 30297871 PMC6218444

[65] Zhou L, Tian S, Qin G. RNA methylomes reveal the m^6^A-mediated regulation of DNA demethylase gene SlDML2 in tomato fruit ripening. Genome Biol. 2019; 20:156. 10.1186/s13059-019-1771-7 31387610 PMC6683476

[66] Begik O, Lucas MC, Liu H, Ramirez JM, Mattick JS, Novoa EM. Integrative analyses of the RNA modification machinery reveal tissue- and cancer-specific signatures. Genome Biol. 2020; 21:97. 10.1186/s13059-020-02009-z 32375858 PMC7204298

[67] Hao H, Hao S, Chen H, Chen Z, Zhang Y, Wang J, Wang H, Zhang B, Qiu J, Deng F, Guan W. N6-methyladenosine modification and METTL3 modulate enterovirus 71 replication. Nucleic Acids Res. 2019; 47:362–74. 10.1093/nar/gky1007 30364964 PMC6326802

[68] Zhao X, Yang Y, Sun BF, Shi Y, Yang X, Xiao W, Hao YJ, Ping XL, Chen YS, Wang WJ, Jin KX, Wang X, Huang CM, et al. FTO-dependent demethylation of N6-methyladenosine regulates mRNA splicing and is required for adipogenesis. Cell Res. 2014; 24:1403–19. 10.1038/cr.2014.151 25412662 PMC4260349

[69] Takahashi T, Nakano Y, Onomoto K, Murakami F, Komori C, Suzuki Y, Yoneyama M, Ui-Tei K. LGP2 virus sensor regulates gene expression network mediated by TRBP-bound microRNAs. Nucleic Acids Res. 2018; 46:9134–47. 10.1093/nar/gky575 29939295 PMC6158488

[70] Hsu SH, Delgado ER, Otero PA, Teng KY, Kutay H, Meehan KM, Moroney JB, Monga JK, Hand NJ, Friedman JR, Ghoshal K, Duncan AW. MicroRNA-122 regulates polyploidization in the murine liver. Hepatology. 2016; 64:599–615. 10.1002/hep.28573 27016325 PMC4956491

[71] Li X, Cassidy JJ, Reinke CA, Fischboeck S, Carthew RW. A microRNA imparts robustness against environmental fluctuation during development. Cell. 2009; 137:273–82. 10.1016/j.cell.2009.01.058 19379693 PMC2674871

[72] Grasso G, Higuchi T, Mac V, Barbier J, Helsmoortel M, Lorenzi C, Sanchez G, Bello M, Ritchie W, Sakamoto S, Kiernan R. NF90 modulates processing of a subset of human pri-miRNAs. Nucleic Acids Res. 2020; 48:6874–88. 10.1093/nar/gkaa386 32427329 PMC7337520

[73] Fernandez N, Cordiner RA, Young RS, Hug N, Macias S, Cáceres JF. Genetic variation and RNA structure regulate microRNA biogenesis. Nat Commun. 2017; 8:15114. 10.1038/ncomms15114 28466845 PMC5418625

[74] Mahony S, Corcoran DL, Feingold E, Benos PV. Regulatory conservation of protein coding and microRNA genes in vertebrates: lessons from the opossum genome. Genome Biol. 2007; 8:R84. 10.1186/gb-2007-8-5-r84 17506886 PMC1929153

[75] Zhou Y, Han C, Wang E, Lorch AH, Serafin V, Cho BK, Gutierrez Diaz BT, Calvo J, Fang C, Khodadadi-Jamayran A, Tabaglio T, Marier C, Kuchmiy A, et al. Posttranslational Regulation of the Exon Skipping Machinery Controls Aberrant Splicing in Leukemia. Cancer Discov. 2020; 10:1388–409. 10.1158/2159-8290.CD-19-1436 32444465 PMC7483384

[76] Jiang L, Huang J, Higgs BW, Hu Z, Xiao Z, Yao X, Conley S, Zhong H, Liu Z, Brohawn P, Shen D, Wu S, Ge X, et al. Genomic Landscape Survey Identifies SRSF1 as a Key Oncodriver in Small Cell Lung Cancer. PLoS Genet. 2016; 12:e1005895. 10.1371/journal.pgen.1005895 27093186 PMC4836692

[77] Li MD, Lou XY, Chen G, Ma JZ, Elston RC. Gene-gene interactions among CHRNA4, CHRNB2, BDNF, and NTRK2 in nicotine dependence. Biol Psychiatry. 2008; 64:951–7. 10.1016/j.biopsych.2008.04.026 18534558 PMC2592606

[78] Yi S, Lin S, Li Y, Zhao W, Mills GB, Sahni N. Functional variomics and network perturbation: connecting genotype to phenotype in cancer. Nat Rev Genet. 2017; 18:395–410. 10.1038/nrg.2017.8 28344341 PMC6020840

[79] Wang W, Kryczek I, Dostál L, Lin H, Tan L, Zhao L, Lu F, Wei S, Maj T, Peng D, He G, Vatan L, Szeliga W, et al. Effector T Cells Abrogate Stroma-Mediated Chemoresistance in Ovarian Cancer. Cell. 2016; 165:1092–105. 10.1016/j.cell.2016.04.009 27133165 PMC4874853

[80] Jiang P, Gu S, Pan D, Fu J, Sahu A, Hu X, Li Z, Traugh N, Bu X, Li B, Liu J, Freeman GJ, Brown MA, et al. Signatures of T cell dysfunction and exclusion predict cancer immunotherapy response. Nat Med. 2018; 24:1550–8. 10.1038/s41591-018-0136-1 30127393 PMC6487502

[81] Fan Q, Ma Q, Bai J, Xu J, Fei Z, Dong Z, Maruyama A, Leong KW, Liu Z, Wang C. An implantable blood clot-based immune niche for enhanced cancer vaccination. Sci Adv. 2020; 6:eabb4639. 10.1126/sciadv.abb4639 32978163 PMC7518870

[82] Cho SY, Chae J, Na D, Kang W, Lee A, Min S, Kang J, Choi B, Lee J, Sung CO, Chuang JH, Lee C, Lee WS, et al. Unstable Genome and Transcriptome Dynamics during Tumor Metastasis Contribute to Therapeutic Heterogeneity in Colorectal Cancers. Clin Cancer Res. 2019; 25:2821–34. 10.1158/1078-0432.CCR-18-3460 30670495 PMC7875093

[83] Hu R, Xu H, Jia P, Zhao Z. KinaseMD: kinase mutations and drug response database. Nucleic Acids Res. 2021; 49:D552–61. 10.1093/nar/gkaa945 33137204 PMC7779064

[84] Angus L, Smid M, Wilting SM, van Riet J, Van Hoeck A, Nguyen L, Nik-Zainal S, Steenbruggen TG, Tjan-Heijnen VC, Labots M, van Riel JM, Bloemendal HJ, Steeghs N, et al. The genomic landscape of metastatic breast cancer highlights changes in mutation and signature frequencies. Nat Genet. 2019; 51:1450–8. 10.1038/s41588-019-0507-7 31570896 PMC6858873

[85] de Miguel FJ, Sharma RD, Pajares MJ, Montuenga LM, Rubio A, Pio R. Identification of alternative splicing events regulated by the oncogenic factor SRSF1 in lung cancer. Cancer Res. 2014; 74:1105–15. 10.1158/0008-5472.CAN-13-1481 24371231

[86] Ghigna C, Giordano S, Shen H, Benvenuto F, Castiglioni F, Comoglio PM, Green MR, Riva S, Biamonti G. Cell motility is controlled by SF2/ASF through alternative splicing of the Ron protooncogene. Mol Cell. 2005; 20:881–90. 10.1016/j.molcel.2005.10.026 16364913

[87] Wan L, Yu W, Shen E, Sun W, Liu Y, Kong J, Wu Y, Han F, Zhang L, Yu T, Zhou Y, Xie S, Xu E, et al. SRSF6-regulated alternative splicing that promotes tumour progression offers a therapy target for colorectal cancer. Gut. 2019; 68:118–29. 10.1136/gutjnl-2017-314983 29114070

[88] Kong J, Sun W, Li C, Wan L, Wang S, Wu Y, Xu E, Zhang H, Lai M. Long non-coding RNA LINC01133 inhibits epithelial-mesenchymal transition and metastasis in colorectal cancer by interacting with SRSF6. Cancer Lett. 2016; 380:476–84. 10.1016/j.canlet.2016.07.015 27443606

[89] Manetti M, Guiducci S, Romano E, Ceccarelli C, Bellando-Randone S, Conforti ML, Ibba-Manneschi L, Matucci-Cerinic M. Overexpression of VEGF165b, an inhibitory splice variant of vascular endothelial growth factor, leads to insufficient angiogenesis in patients with systemic sclerosis. Circ Res. 2011; 109:e14–26. 10.1161/CIRCRESAHA.111.242057 21636803

[90] Chen Y, Huang Q, Liu W, Zhu Q, Cui CP, Xu L, Guo X, Wang P, Liu J, Dong G, Wei W, Liu CH, Feng Z, et al. Mutually exclusive acetylation and ubiquitylation of the splicing factor SRSF5 control tumor growth. Nat Commun. 2018; 9:2464. 10.1038/s41467-018-04815-3 29942010 PMC6018636

[91] Zhou X, Li X, Cheng Y, Wu W, Xie Z, Xi Q, Han J, Wu G, Fang J, Feng Y. BCLAF1 and its splicing regulator SRSF10 regulate the tumorigenic potential of colon cancer cells. Nat Commun. 2014; 5:4581. 10.1038/ncomms5581 25091051

[92] Lee SC, Dvinge H, Kim E, Cho H, Micol JB, Chung YR, Durham BH, Yoshimi A, Kim YJ, Thomas M, Lobry C, Chen CW, Pastore A, et al. Modulation of splicing catalysis for therapeutic targeting of leukemia with mutations in genes encoding spliceosomal proteins. Nat Med. 2016; 22:672–8. 10.1038/nm.4097 27135740 PMC4899191

[93] Lee SC, Abdel-Wahab O. Therapeutic targeting of splicing in cancer. Nat Med. 2016; 22:976–86. 10.1038/nm.4165 27603132 PMC5644489

[94] Agrawal AA, Yu L, Smith PG, Buonamici S. Targeting splicing abnormalities in cancer. Curr Opin Genet Dev. 2018; 48:67–74. 10.1016/j.gde.2017.10.010 29136527

[95] Zhang D, Hu Q, Liu X, Ji Y, Chao HP, Liu Y, Tracz A, Kirk J, Buonamici S, Zhu P, Wang J, Liu S, Tang DG. Intron retention is a hallmark and spliceosome represents a therapeutic vulnerability in aggressive prostate cancer. Nat Commun. 2020; 11:2089. 10.1038/s41467-020-15815-7 32350277 PMC7190674

[96] Sheng J, Zhao Q, Zhao J, Zhang W, Sun Y, Qin P, Lv Y, Bai L, Yang Q, Chen L, Qi Y, Zhang G, Zhang L, et al. SRSF1 modulates PTPMT1 alternative splicing to regulate lung cancer cell radioresistance. EBioMedicine. 2018; 38:113–26. 10.1016/j.ebiom.2018.11.007 30429088 PMC6306353

[97] Gabriel M, Delforge Y, Deward A, Habraken Y, Hennuy B, Piette J, Klinck R, Chabot B, Colige A, Lambert C. Role of the splicing factor SRSF4 in cisplatin-induced modifications of pre-mRNA splicing and apoptosis. BMC Cancer. 2015; 15:227. 10.1186/s12885-015-1259-0 25884497 PMC4399393

[98] Fukuhara T, Hosoya T, Shimizu S, Sumi K, Oshiro T, Yoshinaka Y, Suzuki M, Yamamoto N, Herzenberg LA, Herzenberg LA, Hagiwara M. Utilization of host SR protein kinases and RNA-splicing machinery during viral replication. Proc Natl Acad Sci USA. 2006; 103:11329–33. 10.1073/pnas.0604616103 16840555 PMC1544086

[99] Pilch B, Allemand E, Facompré M, Bailly C, Riou JF, Soret J, Tazi J. Specific inhibition of serine- and arginine-rich splicing factors phosphorylation, spliceosome assembly, and splicing by the antitumor drug NB-506. Cancer Res. 2001; 61:6876–84. 11559564

[100] Kumar A, Coleman I, Morrissey C, Zhang X, True LD, Gulati R, Etzioni R, Bolouri H, Montgomery B, White T, Lucas JM, Brown LG, Dumpit RF, et al. Substantial interindividual and limited intraindividual genomic diversity among tumors from men with metastatic prostate cancer. Nat Med. 2016; 22:369–78. 10.1038/nm.4053 26928463 PMC5045679

[101] Hausser J, Szekely P, Bar N, Zimmer A, Sheftel H, Caldas C, Alon U. Tumor diversity and the trade-off between universal cancer tasks. Nat Commun. 2019; 10:5423. 10.1038/s41467-019-13195-1 31780652 PMC6882839

