# Supplementary material for: Molecular characteristics and clinical implications of serine/arginine-rich splicing factors in human cancer
Source: Aging (Albany NY). 2023 Nov 24;15(22):13287–311. doi: 10.18632/aging.205241 (PMC10713412; doi:10.18632/aging.205241)
Supplement: Supplementary Figures [file aging-15-205241-s001.pdf]

SUPPLEMENTARY FIGURES

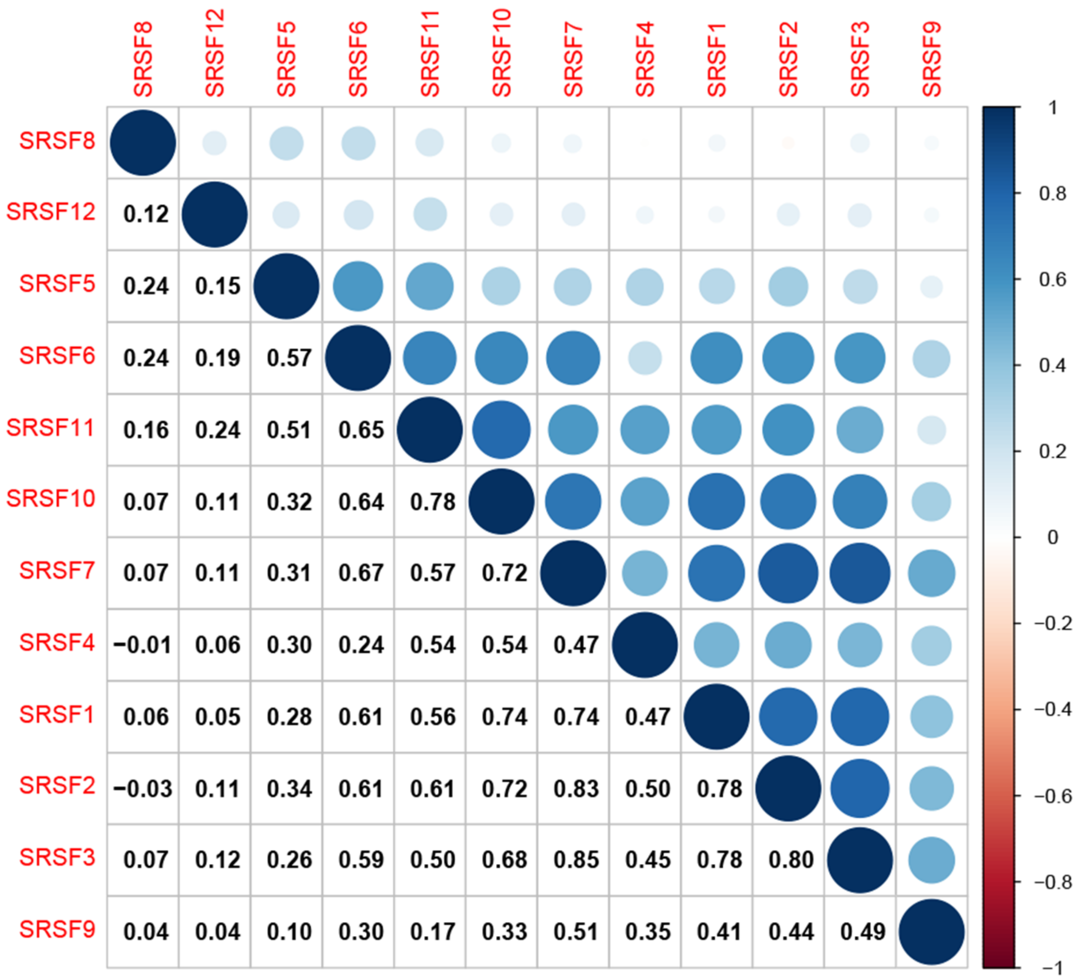

Supplementary Figure 1. Correlation among the SRSFs mRNA expression.

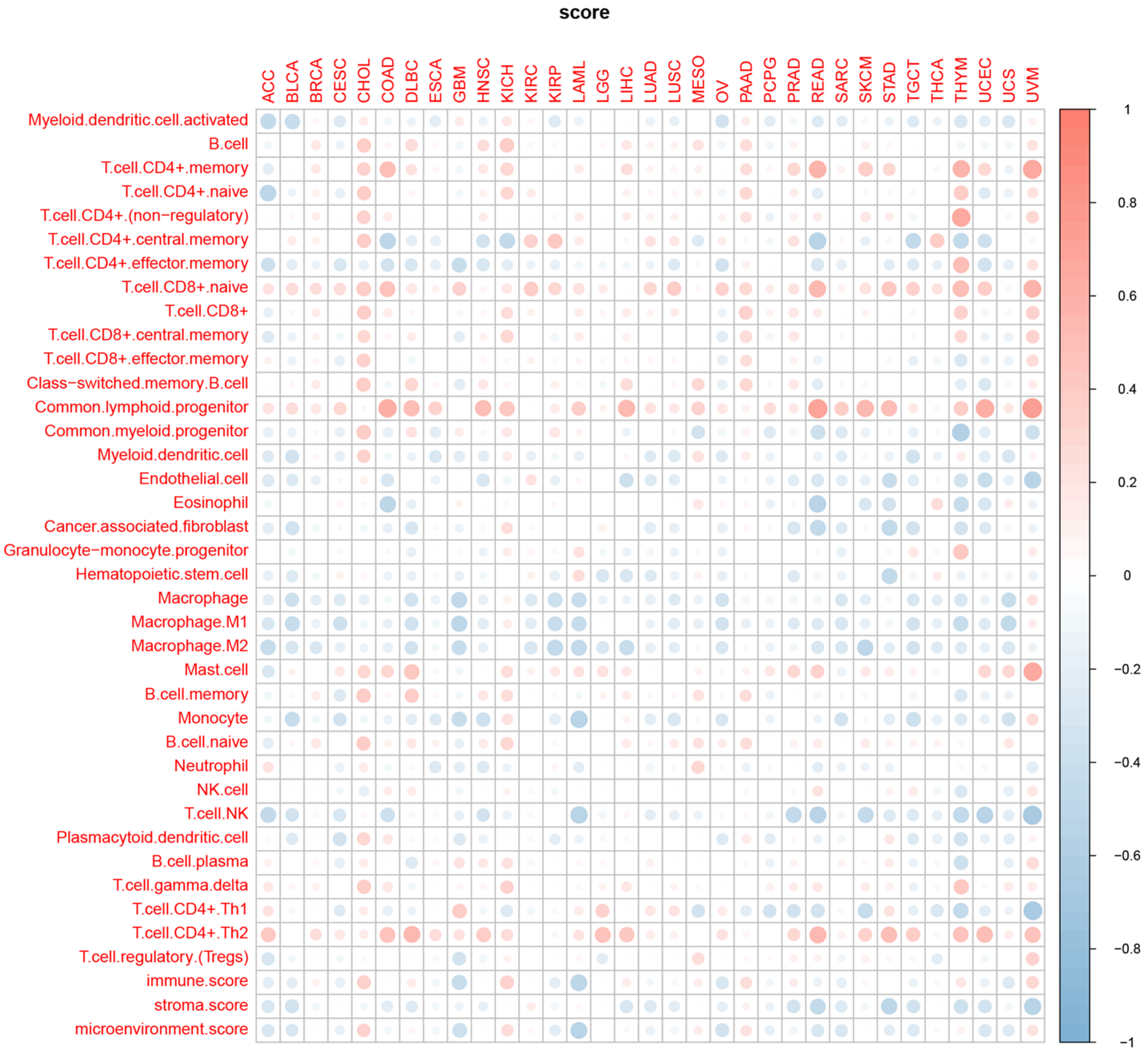

Supplementary Figure 2. Correlation between the SRSFscore and abundance of immune or stromal cells across 33 cancer types. The figure only displayed significant dots with *P*-value <0.05.

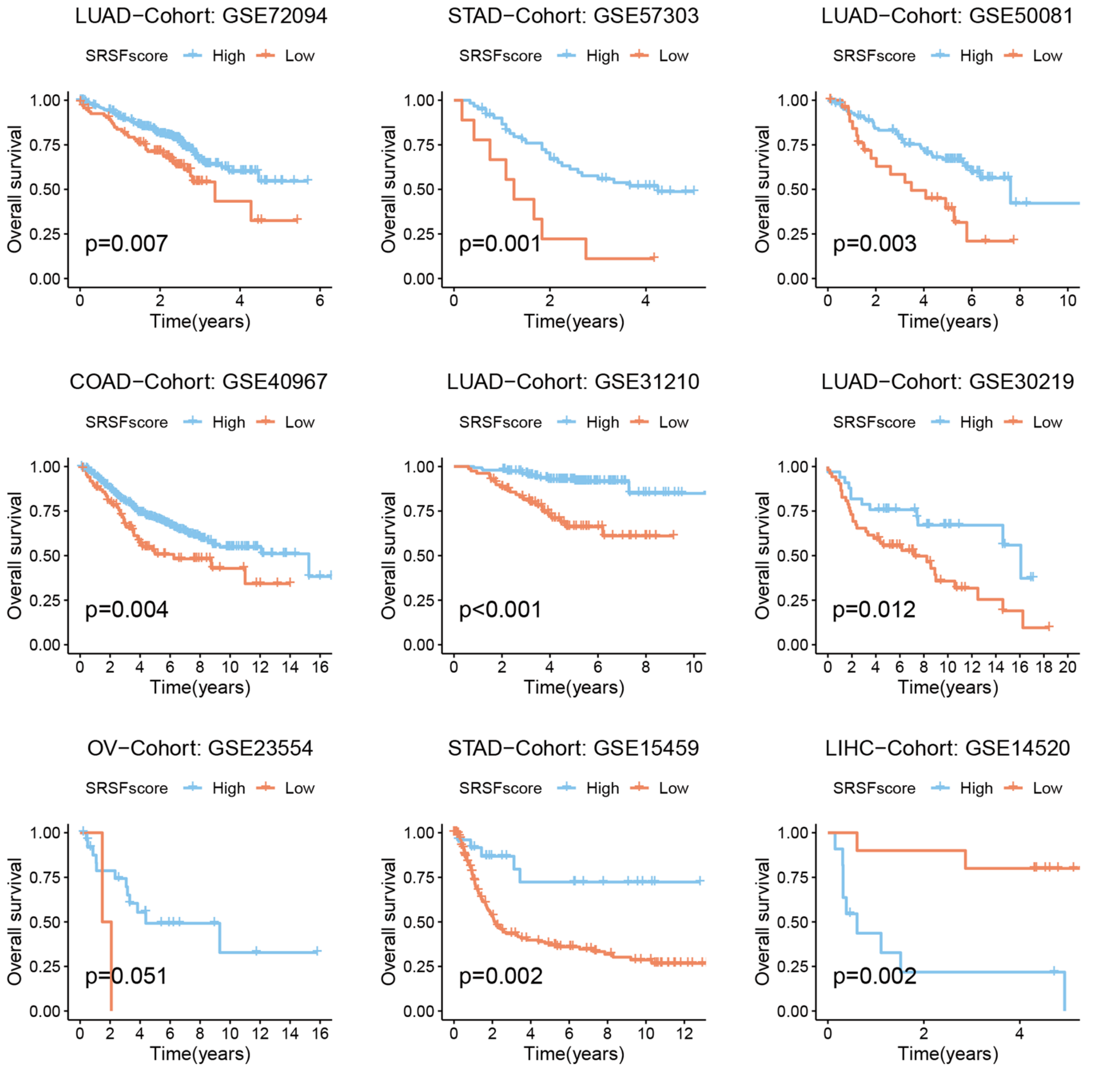

Supplementary Figure 3. Kaplan-Meier survival curves showing the association of SRSFscore to overall survival. Statistical significance was assessed by log-rank test.
